# Translational machinery of the chaetognath *Spadella cephaloptera*: a transcriptomic approach to the analysis of cytosolic ribosomal protein genes and their expression

**DOI:** 10.1186/1471-2148-7-146

**Published:** 2007-08-28

**Authors:** Roxane M Barthélémy, Anne Chenuil, Samuel Blanquart, Jean-Paul Casanova, Eric Faure

**Affiliations:** 1 E.R. Biodiversity and environnement, case 5, Pl. V. Hugo, Université de Provence, 13331, Marseille cedex 3, France; 2 UMR 6540 CNRS DIMAR, Centre d'Océanologie de Marseille, Station Marine d'Endoume, Ch. de la Batterie des Lions, 13007 Marseille, France; 3 Laboratoire d'Informatique, de Robotique et de Microélectronique de Montpellier, UMR 5506, CNRS-Université de Montpellier 2, 161, rue Ada, 34392 Montpellier Cedex 5, France

## Abstract

**Background:**

Chaetognaths, or arrow worms, are small marine, bilaterally symmetrical metazoans. The objective of this study was to analyse ribosomal protein (RP) coding sequences from a published collection of expressed sequence tags (ESTs) from a chaetognath (*Spadella cephaloptera*) and to use them in phylogenetic studies.

**Results:**

This analysis has allowed us to determine the complete primary structures of 23 out of 32 RPs from the small ribosomal subunit (SSU) and 32 out of 47 RPs from the large ribosomal subunit (LSU). Ten proteins are partially determined and 14 proteins are missing. Phylogenetic analyses of concatenated RPs from six animals (chaetognath, echinoderm, mammalian, insect, mollusc and sponge) and one fungal taxa do not resolve the chaetognath phylogenetic position, although each mega-sequence comprises approximately 5,000 amino acid residues. This is probably due to the extremely biased base composition and to the high evolutionary rates in chaetognaths. However, the analysis of chaetognath RP genes revealed three unique features in the animal Kingdom. First, whereas generally in animals one RP appeared to have a single type of mRNA, two or more genes are generally transcribed for one RP type in chaetognath. Second, cDNAs with complete 5'-ends encoding a given protein sequence can be divided in two sub-groups according to a short region in their 5'-ends: two novel and highly conserved elements have been identified (5'-TAATTGAGTAGTTT-3' and 5'-TATTAAGTACTAC-3') which could correspond to different transcription factor binding sites on paralog RP genes. And, third, the overall number of deduced paralogous RPs is very high compared to those published for other animals.

**Conclusion:**

These results suggest that in chaetognaths the deleterious effects of the presence of paralogous RPs, such as apoptosis or cancer are avoided, and also that in each protein family, some of the members could have tissue-specific and extra-ribosomal functions. These results are congruent with the hypotheses of an allopolyploid origin of this phylum and of a ribosome heterogeneity.

## Background

Chaetognaths are a small marine phylum, only comprising about 120 species; they live in various habitats, but most of them are planktonic [1]. They play a key role in marine food webs and are considered to be the second phylum, after copepods in terms of plankton biomass [2]. Thus, they can be considered as a successful phylum. Moreover, Casanova et al. [3], based on more than 20 years of research on the phylum, consider chaetognaths as a model animal. One of us (JPC) described about a quarter of the known species and reported many original observations, such as the progressive stages of acquisition of one pair of appendages on the posterior half of the tail by modifying a part of their balancing fins [4]. He also reported astonishing variations of the secondary muscle, one of the locomotory muscles [5], exhibiting two forms; one is unique in the animal Kingdom (alternation of two sarcomere types) and the other, found only in more or less benthic species, functions by supercontraction.

The phylogenetic position of chaetognaths is always debated. As soon as 1844, Darwin [6] wrote they are "*remarkable for the obscurity of their affinities*." Since then, they have been related to most of the phyla. Nevertheless, for a long time, they were commonly said to be deuterostomes [7,8]. On the basis of anatomical observations on a new "archaic" deep living species he described, Casanova [9] pointed out affinities with molluscs (protostomes). This was an impulsion for new researches. Since a few years, numerous molecular analysis as well as embryological data rejected the relationships with deuterostomes and placed the chaetognath ancestor either at the base of the coelomate Metazoa, just before the splitting protostomes/deuterostomes, or near the protostomes and even as part as protostomes [10-22]. Classical phylogenetic molecular markers such as nuclear rRNA sequences, but also other molecular markers used more recently did not convincingly help to define the Chaetognatha affinities, due to the long branch attraction (LBA) artefact. Mitochondrial investigations using, independent, gene sequences for *Paraspadella gotoi *Casanova, 1990 [16] or *Spadella cephaloptera *Busch, 1851 [15] have both shown close relationships with the protostomes, whereas phylogenetic analyses of these two complete chaetognath mitochondrial genomes in combination have placed chaetognaths either within the Lophotrochozoa or as sister to this clade [21]. Comparisons of mitochondrial gene arrangements also suggested phylogenetic relationships between chaetognaths and Lophotrochozoa [19]. Moreover, recent studies using analyses of expressed sequence tags (EST) encoding ribosomal proteins (RPs) from *Spadella cephaloptera *and (mainly RPs from) *Flaccisagitta enflata *Grassi, 1881 respectively, positioned chaetognaths among protostomes, likely as a sister-group of all other protostome phyla [20] and supported a lophotrochozoan relationship [21]. Fossil evidence suggests that chaetognaths were widespread and diverse in the earliest Cambrian and the difficulties of the phylogenetic position of this phylum is probably partly due to its divergence at an early stage from the primitive ancestor of the Bilateria [22].

The study of molecular evolution requires a battery of genes that are optimally informative at overlapping taxonomic levels [23,24]; for this reason EST analyses and principally RP datasets are particularly useful [20,21]. Ribosomes are the ribonucleoprotein particles responsible for peptide synthesis in all living organisms. As being present in the last universal common ancestor, their basic structural and functional features have been preserved in all diverse descendants. So, the macromolecular components of the ribosome have been useful for evolutionary studies. During translation, the eukaryotic cytosolic ribosome is composed of two subunits: small ribosomal subunit (SSU) and large ribosomal subunit (LSU) consisting of four ribosomal RNA (rRNA) molecules and over 70 associated RPs. The number of cytosolic RPs is around 79 in Eukaryotes and varies very slightly within this clade [25,26]. Moreover, it is widely recognized that in animals a single gene encodes each RP, although most if not all of the RP genes have a number of processed pseudogenes located elsewhere in the genome [27,28]. Contrarily, multiple (often more than two) functional genes encoding each RP are found in plants [29], and in the baker yeast cell (*Saccharomyces cerevisiae*), the 78RPs are encoded by 137 genes and 59 of the genes are duplicated [26]. Interestingly, most of the plants are polyploids [30] and the baker yeast has arisen from ancient whole-genome duplication [31]; it has been suggested that the two divergent classes of both 18S and 28S rRNA genes found in all the extant chaetognaths [32,33] could have arisen from an allopolyploid event (genome combination after species hybridization) [34] which allow us to hypothesize possible presence of RP paralogs in this taxon and ribosome heterogeneity. Moreover, if numerous RP paralog genes are found in chaetognath EST database, this may have consequences for phylogenetic analyses as the use of one form or the other may affect phylogenetic results.

The aim of this study was to analyse RP coding sequences from sequences from a publicly available collection of ESTs from a chaetognath (*S. cephaloptera*) [20] making them available for phylogenetic analysis in bilaterians. As part of our chaetognath genome research concerning genome structure, organization and evolution, here we report 55 complete sequences of cytosolic RPs and data concerning the expression and evolutionary analysis of the corresponding mRNAs.

## Results

### Analysis of the various ribosomal protein multigene families

**Figure 1:**
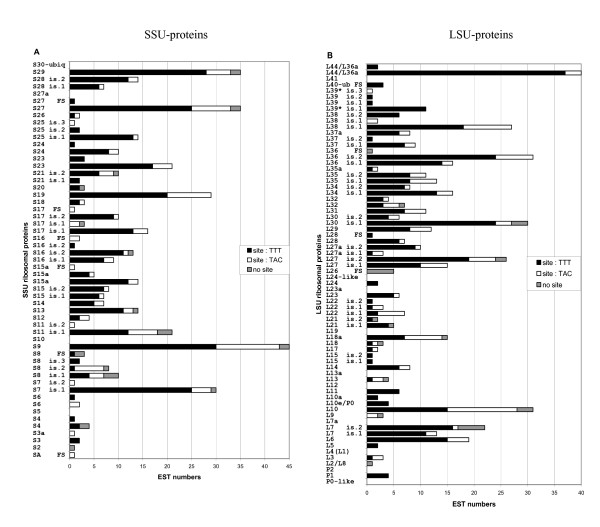
**Frequency of clones sequenced for each cDNA type. A: SSU RP cDNAs, B: LSU RP cDNAs**. It is also indicated when isoforms (is.) have been found and when all the sequences contain frameshift(s) (FS). The characteristics of the 5'-end have been indicated for each cDNA type, TTT potential binding site(s) in black, TAT potential binding site(s) in white and partial sequences which do not contain the 5'-end in grey.

On the 2396 clones representing putative transcripts encoding proteins, 452 clones (18.9%) were identified as representatives of small cytosolic RPs and 511 clones (21.4%) as representatives of large cytosolic RPs. The nomenclature of RPs in different organisms is quite confusing because of many synonyms for the same gene in different organisms; to avoid further confusion, we have followed the nomenclature of the rat [35]. For instance, the SA RP was also known as the 40 kDa RP; RP S3a was also termed v-Fos transformation effector; and S27a and S30 were derived from ubiquitin fusion proteins. The genes were annotated using identity comparisons with the rat RP gene sequences. Bioinformatic analysis of the chaetognath RP cDNAs (DNA complementary to RNA) sequences revealed the candidate clones for 28 SSU RP genes and for 37 LSU RP genes, with respectively between 1 to 45 clones and between 1 to 42 clones for each of the RP gene family (Figure 1 and Table 1).

**Table 1:** Structural characteristics of the complete ribosomal proteins from *S. cephaloptera*

SSU RP name	N° of aa	Mr (Da)	p*I*	N° of EST	EMBL acc. n°	LSU RP name	N° of aa	Mr (Da)	p*I*	N° of EST	EMBL acc. n°
SA	#			1 (FS)		P1	117	11,809	4.24	4	CAL69054
S2	190*			1	CR952433	L2/L8	124#			1	CR953440
S3	250#			2	CR952605	L3	252#			3	CR953541
S3a	210#			1	CR952180	L5	214#			2	CR953623
S4	260	29,417	10.16	5	CAL69092	L6	245	28,213	10.97	19	CAL69056
S6	242#			3	CR953420	L7				35	
S7				31			is.1: 245	28,485	10.73	13	CAL69057
	is.1: 194	22,046	10.16	30	CAL69093		is.2: 245	28,322	10.65	22	CAL69058
	is.2: 194	22,049	10.22	1	CR952582	L9	188	21,489	9.59	3	CAL69059
S8				23		L10	217	25215	10.32	31	CAL69060
	is.1: 208	23,735	10.65	10	CAL69094	L10e/P0	233#			4	CAL69061
	is.2: 208	23,877	10.65	8	CAL69095	L10a	216	24,268	10.04	2	CR952631
	is.3: 208	23,801	10.65	2	CR952624	L11	207	23,095	10.09	6	CAL69062
				3 (FS)		L13	213	24,848	10.75	4	CAL69063
S9	189	22,157	10.62	45	CAL69096	L14	137	15,753	10.65	8	CAL69064
S11				22		L15				2	
	is.1: 156	17,969	10.74	21	CAL69097		is.1: 205	24,092	11.45	1	CR953910
	is.2: 156	17,880	10.64	1	CR952330		is.2: 205	24,146	11.50	1	CR952649
S12	143	15,587	6.34	4	CAL69098	L17	190	21,642	10.45	2	CR952887
S13	151	17,160	10.75	14	CAL69099	L18	188	21,527	11.83	3	CAL69065
S14	151	16,369	10.67	7	CAL69100	L18a	178	20,905	10.73	15	CAL69066
S15				15		L21				7	
	is.1: 144	16,559	10.38	7	CAL69101		is.1: 161	18,655	11.03	5	CAL69068
	is.2: 144	16,529	10.38	8	CAL69102		is.2: 161	18,637	11.33	2	CR953179
S15a	130	14,780	10.12	21	CAL69103	L22				11	
S16				25			is.1: 131	15,025	9.39	10	CAL69068
	is.1: 145	16,345	10.38	14	CAL69104		is.2: 128	14,713	9.59	1	CR953848
	is.2: 146	16,357	10.38	9	CAL69105	L23	140	14,849	10.61	6	CAL69069
				2 (FS)		L24	158	18,033	11.53	2	CR953549
S17				30		L26				5 (FS)	
	is.1: 134	15,568	10.12	19	CAL69106	L27				41	
	is.2: 135	15,742	10.08	10	CAL69105		is.1: 136	15,830	10.53	15	CAL69072
				1 (FS)			is.2: 136	15,723	10.42	26	CAL69073
S18	154	17,829	10.58	3	CAL69108	L27a				13	
S19	139	15,514	10.49	29	CAL69109		is.1: 145	16,073	10.82	3	CAL69072
S20	125	13,634	10.04	3	CAL69110		is.2: 145	16,065	10.57	10	CAL69073
S21				12		L28	132	14,440	11.85	8	CAL69074
	is.1: 81	8,773	7.58	2	CR953395	L29	83	9,604	11.87	12	CAL69075
	is.2: 83	9,077	7.58	10	CAL69111	L30				36	
S23	143	15,779	10.76	24	CAL69112		is.1: 114	12,381	9.74	30	CAL69076
S24	135	15,383	10.88	11	CAL69113		is.2: 114	12,411	9.79	6	CAL69077
S25				17		L31	123	14,044	10.87	11	CAL69078
	is.1: 115	12,719	10.12	14	CAL69114	L32	133	15,693	11.46	11	CAL69079
	is.2: 114	12,663	10.15	2	CR953826	L34				24	
	is.3: 127	13,911	10.50	1	CR953802		is.1: 130	14,470	11.42	16	CAL69080
S26	106	12,011	10.73	2	CR953685		is.2: 131	14,547	11.42	8	CAL69081
S27	84	9,273	9.20	36	CAL69115	L35				24	
S28				21			is.1: 123	14,326	11.72	13	CAL69082
	is.1: 64	7,279	10.54	7	CAL69116		is.2: 123	14,468	11.49	11	CAL69083
	is.2: 64	7,279	10.54	14	CAL69117	L35a	135	15,397	10.96	2	CR953126
S29	56	6,393	9.93	35	CAL69118	L36				49	
							is.1: 106	12,318	11.49	16	CAL69084
							is.2: 105	12,223	11.25	32	CAL69085
										1 (FS)	
						L37				10	
							is.1: 100	11,621	11.77	9	CAL69086
							is.2: 100	11,561	11.77	1	CR952772
						L37a	93	10,411	11.04	8	CAL69087
						L38				35	
							is.1: 70	8,220	10.43	29	CAL69088
							is.2: 70	8,198	10.64	6	CAL69089
						L39				15	
							is.1: 51	6.321	12.55	13	CAL69090
							is.2: 51	6.315	12.55	1	CR953375
							is.3: 51	6.305	12.55	1	CR953100
						L40-ubiq				3 (FS)	
						L44/L36a	106	12,400	10.67	42	CAL69091

Accession numbers correspond to protein sequences, except when less than three EST sequences have been found, where EST accession numbers are been given. When two ESTs belonging to the same mRNA type have been found, only the accession number of the longest sequence has been given. Abbreviations: Da, Daltons; FS, all the sequences from a cDNA type contain frameshift(s); L40-ubiq, L40-ubiquitin; Mr, molecular weight; pI, isoelectric point. # Incomplete COOH-end; *Incomplete NH_2_-end.

**Figure 2:**
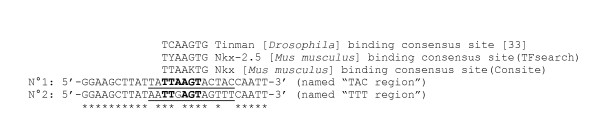
**Alignment of the two consensus 5'-ends of the *S. cephaloptera *ribosomal protein genes**. The 28 nucleotides region named n°2 has been found in 249 ESTs putatively encoding for 28 different SSU RPs, and in 319 ESTs putatively encoding for 37 different LSU RPs. The 28 nucleotides region of the sequence n°1 has been found in 75 ESTs putatively encoding for 25 different SSU RPs and in 96 ESTs putatively encoding for 29 different LSU RPs. The stars (*) indicate nucleotides which are conserved between these two sequences. The nucleotide regions which differ between these two sequences have been underlined and have named respectively TAC consensus site and TTT consensus site. The nucleotides which are conserved between these two consensus sites and Tinman/Nkx2.5 binding consensus sites are indicated in bold letters; K representing T or G.

Eukaryotic RP genes appear to belong to multigene families. However, contrarily to fungi or plants, in animals, only one gene from each family is usually transcriptionally active; almost all the remainders of the genes are inactive pseudogenes [27,28]. Surprisingly, in the chaetognath EST database, at least half of the RPs appeared to have two, or sometimes more, types of mRNA (isoforms in Figure 1). Within a gene family, the percentage of identity of the various members at the nucleotide level varies from 61 to 88 % (data not shown). In addition, one type of mRNA is almost always overrepresented compared to the other(s). In approximately half of the cases, for a RP gene family (for example S15), several mRNAs are found in this EST database, these mRNAs can be divided in two or more types and each type encodes the same isoform (in this case : S15 isoform 1 for S15 mRNAs of the type 1 and S15 isoform 2 for S15 mRNAs of the type 2); moreover, generally, within each type, the mRNA sequences can be divided in two subtypes, which differ principally by a short sequence in their 5'-ends ("TTT" and "TAC" sites in Figures 1 and 2). Indeed, in the clones containing a putative entire mRNA leader sequence, most of them bear the region 5'-TAATTGAGTAGTTT-3' (named TTT) or a region highly homologous (74.5% and 74.8 % of the clones for, respectively, SSU and LSU RP genes) while the others bear the region 5'-TATTAAGTACTAC-3' (named TAC) or a region highly homologous (25.5% and 25.2% of the clones for respectively, SSU and LSU RP genes) (Table 2). According to us, these two regions, which have 10 nucleotides after the potential transcriptional initiation base, could contain binding site(s) for transcription factor(s). Indeed, in some genes, DNA binding site(s) have been identified in the downstream region of the transcriptional start site [36,37]. In addition, it is well known that most RP genes have common promoters and are therefore assumed to have a unified gene expression control mechanism [38]. For these two reasons, we have scrutinized the 5'-ends of the RP mRNAs for potential transcription factor binding sites using two prediction programs (ConSite, TFSEARCH). Although no strict consensus sequence can be identified, the bioinformatic analyses reveal an interesting feature, i.e., the 5' part of the regions described above could constitute a binding site for a member of the Tinman-Nkx2.5-Csx homeodomain factor family [39] (Figure 2). Interestingly, one of the putative binding site (TAC) is more similar to the consensus binding site than the other (TTT) and increasing evidence indicates that individual Nkx factors are critical regulators of whole organ development [40], suggesting a putative role of these regions in development. On another hand, the 3' parts of TTT and TAC regions exhibit great nucleotidic differences after the putative Tinman site (i.e, TT and AC respectively), suggesting that these sequences could also bind some specific protein factors, but our bioinformatic analyses do not allow to identify putative candidates. Moreover, in only one case, mRNA subtypes [mRNA sequences which differ principally by the 5' untranslated region (5'UTR)] belonging to the same type do not encode exactly the same amino acid sequence; L39 isoform-3 differs from the L39 isoform-1 by only one amino acid, but however, only one EST encoding the isoform-3 has been found (Figure 1 and Table 3).

**Table 2:** Frequency of the TTT and TAC regions in the chaetognath ribosomal protein cDNAs with complete 5'-ends

Ribosomal subunit	TTT putative binding site (5'-TAATTGAGTAGTTT-3')		TAC putative binding site (5'-TATTAAGTACTAC-3')	
	% of clones bearing this sequence	% of clones bearing a highly homologous sequence	% of clones bearing this sequence	% of clones bearing a highly homologous sequence

SSU RP genes	63.7	10.8	21.8	3.7
LSU RP genes	70.3	4.5	19.8	5.4

The percentage of identity between consensus sequences and highly homologous sequences is always ≥ 69%.

**Table 3:** Search of differences in biologically significant sites between chaetognath ribosomal protein isoforms using Prosite

Protein name	Isoform number	Aa numbers	EST numbers	% identity/overall similarity	Motifs which are different
*SSU ribosomal proteins*					

S7	1	194	30	82.0/97.9	cAMP:1 – PKC:3
	2	194	1		cAMP:2 – PKC:6
S8	1	208	9	is.1/is.2: 96.6/100	amidation:2 – cAMP:2 – myristil:2 – nuclear:3 – PKC:6
	2	208	8	is.2/is.3: 97.1/100	amidation:1 – cAMP:4 – myristil:2 – nuclear:4 – PKC:8
	3	208	2	is.1/is.3: 97.1/100	amidation:2 – cAMP:4 – myristil:1 – nuclear:4 – PKC:7
S11	1	156	24	98.1/99.4	nuclear:1
	2	156	1		nuclear:0
S15	1	144	7	99.3/100	N.D.
	2	144	8		N.D.
S16	1	146	9	93.8/96.6	CK2:2
	2	145	13		CK2:3
S17	1	134	19	87.4/96.3	CK2:0 – myristil:0 – nuclear:1 – sulfation:1
	2	135	10		CK2:1 – myristil:1 – nuclear:0 – sulfation:0
S21	1	81	2	83.1/92.8	cAMP:1 – myristyl:2 – PKC:3 – tyr:0
	2	83	10		cAMP:2 – myristyl:1 – PKC:2 – tyr:1
S25	1	115	14	is.1/is.2: 82.8/93.9	cAMP:1 – CK2:0 – myristil:2 – nuclear:1
	2	114	2	is.2/is.3: 71.7/83.5	cAMP:1 – CK2:1 – myristil:1 – nuclear:1
	3	127	1	is.1/is.3: 73.2/83.5	cAMP:0 – CK2:0 – myristil:2 – nuclear:0
S28	1	64	8	98.4/100	N.D.
	2	64	14		N.D.

*LSU ribosomal proteins*					

L7	1	245	13	85.7/95.5	cAMP:1 – PKC:2
	2	243	23		cAMP:0 – PKC:3
L15	1	206	1	98.1/99.0	N.D.
	2	207	1		N.D.
L21	1	161	5	97.5/88.1	myristil:2 – PKC:4
	2	161	2		myristil:1 – PKC:5
L22	1	121	10	81.2/92.1	amidation:1 *- *CK2:2- myristil:4
	2	128	1		amidation:0 *- *CK2:1- myristil:2
L27	1	136	15	88.2/99.2	myristil:0 – PKC:2
	2	136	26		myristil:1 – PKC:3
L27a	1	145	3	89.6/97.2	CK2:1
	2	145	10		CK2:0
L30	1	114	20	96.5/98.2	PKC:4
	2	114	6		PKC:5
L34	1	130	16	95.4/97.7	cAMP:3 – PKC:1
	2	131	8		cAMP:4 – PKC:2
L35	1	123	13	89.4/98.4	tyr:0
	2	123	11		tyr:1
L36	1	105	16	90.6/98.1	PKC:4
	2	106	32		PKC:2
L37	1	100	9	99.0/99.0	N.D.
	2	100	8		N.D.
L38	1	70	29	92.9/100	asn:1 – CK2:1 – PKC:3
	2	70	5		asn:0 – CK2:0 – PKC:2
L39	1	51	13	is.1/is.2: 96.1/98.0	myristil:0
	2	51	1	is.1/is.3: 98.0/98.0	myristil:1
	3	51	1	is.2/is.3: 94.1/96.1	myristil:0

Within a protein family, each of the isoforms are putatively encoded by different types of cDNA, except the L39 isoform-1 and isoform-3 which are encoded by cDNAs belonging to two different subtypes (TTT and TAC respectively) of a same type. Abbreviations: amidation, amidation site; cAMP, cAMP- and cGMP-dependent protein kinase phosphorylation site; CK, casein kinase II phosphorylation site; myristil, N-myristoylation site ; N.D., no difference; nuclear, bipartite nuclear targeting sequence; PKC, protein kinase C phosphorylation site ; tyr, tyrosine sulfation site.

### *Spadella cephaloptera *ribosomal proteins from the small (SSU) and large (LSU) subunits

SSU of eukaryotic ribosomes contain generally 32 proteins [27]. We have identified ESTs encoding complete open reading frames (ORFs) for 23 on 32 SSU RPs in the chaetognath database. Unfortunately, sequences of S2, S3, S3a and S6 proteins are incomplete; due to frameshifts, the sequence of SA protein can not be obtained and no ESTs encoding for S5, S10, S27a and S30-ubiquitin-like proteins have been found.

Generally 47 different proteins, including 2 short polypeptides, are present in LSU of eukaryotic ribosomes, but in some taxa this number can reach 50. We have identified ESTs encoding complete ORFs for 32 on 47 LSU proteins in chaetognath EST database. Four sequence proteins are incomplete (L2/L8, L3, L5, L10e/P0); due to frameshifts, the sequence of L6 protein cannot be obtained and ESTs encoding for P0-like, P2, L4(L1), L7a, L12, L13a, L19, L23a, L24-like and L41 proteins are missing. For the L41 mRNA, it is probably due to its short length, since the polypeptide is only 25 amino acids long in rat. Numerous characteristics of *S. cephaloptera *RPs are given in Table 1: number of amino acids, calculated molecular weight (Mr) and calculated isoelectric point (pI).

### Comparative analysis of the isoforms of ribosomal proteins

Interestingly, generally in the chaetognath EST database, when two or more types of mRNA putatively encode a RP, their deduced amino acid sequences differ (Figure 1 and Table 3). In addition, in approximately half of the cases, the number of deduced amino acids between two isoforms belonging to the same RP family is different (third column in Table 3). Moreover, no alternatively spliced transcripts have been identified. On the other hand, these isoforms are probably not due to cloning, PCR and/or sequencing artefacts, because, generally for a RP type, numerous cDNA sequences are strictly similar and when nucleotide differences are found, the integrity of the ORF and of the TTT or TAC regions are generally conserved; in more than half of the cases, within a same RP family, for each proteic member, the number of corresponding mRNA types is higher than three. In addition, for 9 RP families, the isoforms shared less than 90% identity at the amino acid level (column 5 in Table 3). Indeed, for all the RP families, the biologically significant sites were predicted in the deduced amino acid sequences using the program PROSITE. Interestingly, according to these criteria, most of the isoforms exhibit one or more differences suggesting various putative physiological roles (column 5 in Table 3).

### PCR evidence of paralogous genes

As the EST library has been constructed using several individuals [20], inter-individual variations could not be excluded. Using specific PCR primers of all the members of 4 RP gene families, we have evidenced that most of the paralogous genes could be isolated even from single individual DNA preparations (Table 4). Similarly, PCR using primers in the putative transcription factor binding sites show that both classes of genes are present in the chaetognath genome. Some PCR amplifications gave negative results; these concern, except for S8 isoform 3, cases where no EST has been found in the *S. cephaloptera *library. On the other hand, numerous paralogous genes for which no ESTs have been obtained in *S. cephaloptera *library are present in the chaetognath genome; this principally concerns the genes bearing the putative TAC transcription binding sites which were probably under-expressed in the juvenile chaetognaths used for the library construction.

**Table 4:** Presence of members of 4 ribosomal gene families in three *S. cephaloptera *individuals using PCR

			ESTs with TTT sites					ESTs with TAC sites			
			
RP	Is.	Name of the reverse primers (5'-3')	EMBL acc. n° of ESTs which bear the primer sequences	EMBL acc. n° of ESTs with 1 or 2 internal mutations in the primer sequences	PCR results by individual			EMBL acc. n° of ESTs which bear the primer sequences	PCR results by individual		
					1	2	3		1	2	3

S8	1	S8-1R	CR952841, CR953486	CR952238, CR953840	+	+	+	CR952310, CR953241	+	+	+
	2	S8-2R	CR952852, CR953783	none	+	+	+	CR952219, CR953331, CR952138, CR952189	+	+	+
	3	S8-3R	CR952624, CR952678	none	-	-	-	none	-	-	-
S25	1	S25-1R	CR952125, CR952226, CR952356, CR952457, CR952866, CR953401, CR953717, CR953720, CR954024, CR954106	CR952061, CR952409, CR953636	+	+	+	CR953753	+	+	+
	2	S25-2R	CR954093, CR953826	none	+	+	-	none	-	-	-
	3	S25-3R	none	none	-	-	-	CR953802	-	-	+
L15	1	L15-1R	CR953910	none	+	+	-	none	+	-	+
	2	L15-2R	CR952649	none	+	-	+	none	-	-	+
L27a	1	L27a-1R	CR952520, CR952575, CR953281, CR953937	CR952087, CR952954, CR953676	+	+	+	CR953609	+	+	+
	2	L27a-2R	none	CR952757	+	+	+	CR952635, CR953421	+	+	+

For all the members of these four gene families, no internal mutations in the regions bearing the putative TAC sites have been found. Abbreviations and symbols: none, no EST clone has been found in the *S. cephaloptera *library; RP, ribosomal protein; is., isoform; Acc. n°, EMBL accession numbers; +, PCR with positive results; -, PCR with negative results.

### Phylogenetic trees of ribosomal proteins

**Figure 3:**
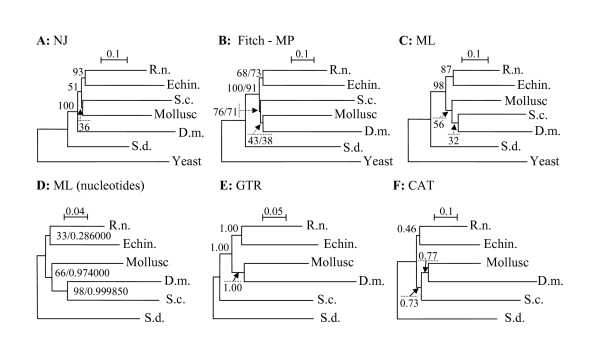
**Phylogenetic trees of the selected ribosomal proteins sequences (see Table 2 and Methods)**. The trees A, B and C were obtained using respectively Neighbor Joining (NJ), Fitch, Maximum Parsimony (MP), and Maximum Likehood (ML) methods on an amino acid dataset. The trees constructed using Fitch and MP methods have a similar topology. In D, the ML tree using the first two codon positions and the model selected by MrAIC, GTRIG, ML estimated base frequency, a gamma (2) distribution for site substitution rates, and an estimated proportion of invariant sites. Similar topologies were obtained with ML using codon models and with a non homogeneous non stationary ML method allowing G+C equilibrium frequency to vary (see text). Trees E and F were obtained using respectively the GTR model with a MCMC bayesian method and the CAT mixture model on an amino acid dataset. Numbers indicate bootstrap values or branch support; in tree B, MP and Fitch values are respectively at the left and at the right, in tree D, after the slash, the aLRT (actually the minimum of the CHI2-based parametric and non parametric aLRT estimated value). Abbreviations: D.m., *D. melanogaster*; Echino., Echinoderm; R.n., *R. norvegicus; *S.c., *S. cephaloptera; *S.d., *S. domuncula*; Yeast, *S. cerevisiae*.

Phylogenetic trees from the multiple alignments of 7 amino acid mega-sequences constructed using the MP, ML, NJ and Fitch methods are in Figure 3a, b, c. All four methods applied on the seven taxa datasets differ only by the position of protostomian and chaetognath sequences which are never strongly supported, but all topologies show chaetognaths belonging to protostomes, however, this is not statistically supported (the best bootstrap value, 76 %, is for the Fitch analyses). The monophyly of Bilateria is well supported in all the analyses (bootstrap values > 91%). The monophyly of Deuterostomia is always recovered with bootstrap values > 68%.

More advanced ML analyses were investigated on a nucleotide dataset from 6 taxa, and the best model selected by MrAIC, on first and second codon positions, is in Figure 3d. In these analyses where the sponge was the outgroup, we consistently found a clade joining the chaetognath and *Drosophila *(Arthropoda), generally with very high bootstrap values (from 83 to 100%), embedded within a larger clade including the mollusc sequences, and we also generally found a deuterostomian clade joining the rat and the echinoderm. The changes observed among the distinct codon position partitions only slightly affected bootstrap values, not topology. The use of codon models also yielded the same topology, as well as the use of the non stationary model allowing G+C contents to vary among lineages. Relative rate tests with respect to the sponge outgroup revealed that the chaetognath and the mollusc had very significantly distinct evolutionary rates, suggesting that the union of the fast-evolving Drosophila and the fast-evolving chaetognaths may be an LBA artefact (see Figure 3d). Base composition also appears biased and susceptible to artefactually join the chaetognath and the *Drosophila *sequences which are the most G-C rich, with G+C levels for second and third codon positions, of 79.1% – 58.3% for *Drosophila*, 71.3 % – 54.9% for the chaetognath, 68.7% – 54.1% for the echinoderm, 55.2% – 47.8% for the mollusc, 60.9% – 51% for the rat, and 47.5% – 46.3% for the sponge. The best model of evolution selected by MrAIC and used for subsequent ML analyses was the most parameter rich and general one, GTR-IG-General Time Reversible model with some invariant sites, and variation of evolutionary rates among sites. The selection of such a parameter rich model is not surprising given the high number of sites. The input of a user tree, where we placed the chaetognath at the base of a protostomian clade did not result in a different topology or branch support than when a starting tree is inferred via BIONJ (for PhyML analyses).

For bayesian analyses on amino-acid sequences, the phylogenies inferred with the GTR and CAT models differ in the placement of the chaetognath, and in the estimated posterior supports of clades (Figure 3e, f). Under GTR, the chaetognath and the sponge sequences cluster together with a strong support (Figure 3e, but posterior bayesian probabilities are often strong), whereas under CAT, it clusters with Protostomia (Figure 3f). In spite of displaying low support for clades, the CAT topology is identical to that previously found by Marletaz et al. [20], using more species and focusing on the chaetognath phylogeny. Both models obtained the deuterostomian monophyly by grouping together rat and echinoderm but with a low posterior support in CAT analyses (46%).

## Discussion

Generally, in EST databases, multiple sequences encoding the same RP have been found, because RPs are well expressed and their mRNAs are abundant and over-represented in the cDNA library. In addition, random sequencing of cDNAs on a large scale always results in high number of sequences encoding RPs [41]. As expected, in the *S. cephaloptera *EST database, in spite of the relatively low number of clones which represent transcripts encoding proteins (only 2396), we were able to deduce complete amino acid sequences of 55 RPs, probably because a) in this cDNA library, inserts with a size greater than 800 bp have been selected, b) although only the 5'-ends of the clones from this library has been sequenced, the generally short length of the RP mRNAs allow to obtain complete sequences, c) 40.3% of the clones encoding proteins are RP mRNAs, and d) most of the RP genes (61%) were indeed found on EST clusters composed of 4 or more sequences. The two extreme examples are S9 and L44/L36a proteins, which were respectively encoded on 45 and 42 ESTs (Table 2). However, we have found only 1 EST for SA, S2, S3a and L2/L8, and if the ORF contains frameshift(s) and/or if it is partial, it becomes impossible to obtain the complete protein sequence. In addition, ESTs encoding for 14 RPs are missing. It is not surprising for two of these proteins, P0-like which is missing in rat and L24-like which is probably not associated with ribosome; for the others, it is probably due to a bias in the EST database. The reason for the huge disproportion in numbers of ESTs encoding the different RPs in *S. cephaloptera *database could also be just the consequence events such as mRNAs stability/instability, differences in efficiencies of mRNAs copying and insertions of cDNAs in the Lambda-phage.

Eukaryotic RP genes appear to belong to multigene families [27,28]; however, great differences have been found between the different Kingdoms. In the yeast cell, where approximately half of the RP genes are duplicated [26], in all cases, both gene copies are transcribed although their expression levels often differ considerably [42]; moreover, the proteins encoded by duplicated genes have identical or virtually identical sequences and are functionally indistinguishable. In plants, multiple functional RP isoforms could be produced [43]. In contrast, generally, in animals only a single gene encodes each RP, the other members of each gene family are pseudogenes [27]. Consequently, analyses of animal EST databases reveal that RP appeared to have only one type of mRNA; the exceptions are rare, for example, in the channel catfish (*Ictalurus punctatus*) EST analysis has revealed, except for three protein types, that each other RP type is encoded by only one type of mRNA [44,45]. Surprisingly, analysis of *S. cephaloptera *EST database reveals a more complicated pattern; almost each isoform could be encoded by two mRNA subtypes which only differ by a short region in their 5'-end sequences; moreover, in approximately half of the RP gene families, deduced isoforms have been found and the generally relatively high number of clones suggest that the corresponding mRNAs are probably translated and the proteins functional. These two events complicate the comprehension of the molecular evolutionary history of chaetognaths. Moreover, PCR experiments have evidenced that at least for 4 ribosomal gene families, the various genes variations are intraindividual variations (Table 4).

Within a mRNA type, generally, the sequences could be divided in two subtypes (named TTT and TAC) which differ by a short region in the 5'-UTR. These regions could correspond to two different transcription factor binding sites. In *S. cephaloptera*, as each of the many entire RP cDNAs has only one of the putative binding sites, this means that the two subtypes could have differential transcription patterns, whereas in other taxa, this feature is restricted to a low number of RP genes. As differences in promoters generally correspond to diverse RP gene expression control in specific tissues [38], our results suggest that one of the putative promoter site (probably TTT, which yields more mRNA) plays certainly a role during housekeeping conditions, whereas the other site (TAC, which yields less mRNA) would allow an expression of RPs when a very large quantity of RPs would be essential in specific tissues and/or in the most crucial development stages. In addition, almost all the RP genes contain one of the two putative binding sites. Two other elements are in favour of this hypothesis; one is that two subtypes for a given gene family encode identical proteins except in one case; the second element is that bioinformatic prediction suggests that one of the 5'-end region could constitute a binding site for members of an homeobox factor family members which are tissue-specific transcription factors and are critical regulators of whole organ development [40].

The analysis of the chaetognath EST database has also revealed a relatively great number of RP paralogs. If some of them, with a low number of ESTs, could be artefacts, the others, with a higher number of ESTs (Table 3), could have a physiological significance. Indeed, the identical or similar sizes of the paralogous members of each protein families added to cDNA analyses evidenced that these isoforms are not due to expression of differentially spliced mRNAs. Moreover, bioinformatical analyses suggest that most of the isoforms exhibit differences in their biologically significant sites (column 5 in Table 3). RP isoforms have been found in other animal taxa. However, the numbers of paralogs is lower; for example, in the channel catfish, if exclude alternative spliced transcripts which concern only the S3 family, paralogs have been found for only two types of RPs (S26 and S27) and one paralog pair has a high percentage of amino acid similarity with 94.8% identity, whereas the other paralog pair only differs by one amino acid [44,45]. In human, two RP genes on the sex chromosomes, one on the Y and one on the X, are both widely transcribed in human tissues and encode two isoforms of S4 RP which differ at 19 of the 263 amino acids [46]. In addition, two genes encoding different L39 proteins have also been evidenced in human [47]. In rat, two functional genes are reported for S27; multiple transcripts encode each isoform and exhibit different tissue expression patterns [48]. Moreover, in sponge (*Suberites domuncula*), no RP isoforms have been evidenced [41]. To our knowledge, except chaetognaths, the presence of numerous RP isoforms has only been evidenced in plants ([43] and references therein). For example, due to the extensive segmental duplication of the *Arabidopsis *genome, all its RP genes have between two and several paralogs; and assessing RP gene expression by the presence of an EST showed that at least 77% of RP genes (not including the 21 genes with incomplete ORFs) are expressed at a level detectable by an EST [49].

The roles of multiple functional RP isoforms in plants remain unclear although it has been proposed that expression of multiple RP genes from a single family may be necessary to accommodate high – or specific – translational needs in growing plant tissue; thus, RP genes copies under development regulation may be required in addition to those gene copies that are constitutively expressed [29,49]. For example, *Arabidopsis *RP gene L16 is present as two copies in the genome, with one isoform expressed in proliferating tissues and the other expressed in more specific tissues [50]; similarly, differential transcriptional regulation of the two RP L23A genes has also been reported [51]. Moreover, differential expression of homeologous (genes duplicated by polyploidy) 18S-5.8S-26S rRNA genes has been shown in plant allopolyploids [52] and expression of multiple genes in a RP gene family may be indicative of ribosome heterogeneity [53]. Surprisingly, chaetognaths exhibit numerous molecular analogies with plants; two classes of paralogs of 18S-28S rRNA have been reported [32,33], which could be the result of an allopolyploid event in the ancestor of all the extant chaetognaths [34]. Moreover, in *S. cephaloptera*, one of the 18S class plays a ubiquitous role whereas the other is specific to oocytes [54]. The great number of RP paralogs in this species could be the result of the allopolyploidy and we hypothesize that two populations of ribosome could exist in chaetognath cells; one of them contains the housekeeping rRNA (Class I) and the isoforms for which numerous mRNA have been found in the EST database and which give relatively short branches in phylogenetic reconstructions (data not shown); the other contains the class II rRNAs with the other isoforms. Moreover, a preliminary observation suggests that, in chaetognaths, the positive or negative selection of RP families which contain paralogs has probably some functional reasons. Indeed, in *Escherichia coli*, it has been evidenced that most RP genes are crucial for ribosome assembly or functionality, such as proteins implicated in the early assembling proteins (S4, S7, S8, S15, S17, L2, L3, L4, L5, L15, L18), the bridges between two subunits (S13, S15, S19, L2, L5, L14), contact with tRNA (S7, S9, S12, S13, L1, L5), and the surrounding polypeptide exit channel (L22, L24, L29) [55]. It is interesting to compare this list of proteins to those given for the chaetognath putative isoforms (Table 3); only S7, S8, S15, S17, L15 and L22 are present in the two lists (i.e., have isoforms and fit the above functions). In addition, for S7, L15 and L22, a paralog is encoded by only a unique clone (EST = 1 in Table 3), suggesting possible sequencing artefacts, and the two S15 paralogous proteins have 100 % of similarity. Therefore, we hypothesize that paralogs for "crucial RPs" could be strongly unfavourable. If RP paralogs which exhibit various non ribosomal functions were to interact with the ribosome, this could induce an inactivation of the translation mechanism. Contrarily, if this event occurs with other non crucial RPs, it could be selectively neutral.

Alternatively, expression of multiple gene family members may also be indicative of multiple functions for RPs from any given gene family, with some members having ribosomal functions and other extraribosomal roles. It is well known that many RPs perform additional extra-ribosomal functions in cells. In mammalian, where the number of RP paralogs is very low, RPs also exhibit various secondary functions in DNA repair, apoptosis, drug resistance and proliferation. They are involved in different cellular processes, from replication and regulation of cell growth to apoptosis and malignant transformation [56,57]; and consequently the expression of their genes could vary considerably [58,59]. In addition, zebrafish carrying heterozygous mutations in a number of RPs are predisposed to cancer [60]. According to us, probably when two or more paralogous RPs exhibit several differences in their primary sequences, one of the paralog plays its "conventional role" as component of ribosome, while the other(s) perform(s) extra-ribosomal functions. Moreover, it has been proposed that gnathostomes had undergone two events of polyploidization leading to octaploidy [61] and in this clade, in each RP gene family, generally a single gene is functional, suggesting that in each RP gene family, all the paralogs but one are subject to strong counter-selection; this is not the case in chaetognaths, putatively allopolyploids, which could have overcome the deleterious effects of paralog RPs. Interestingly, in *S. cephaloptera*, in more half of the RP paralog families, the percentage of identity between the members of each family is less than 93% (Table 3); this could correspond to a subfunctionalization, after ploidy, the homoeolog copies specialize to perform complementary functions [62,63]. A great number of RP paralogs generate another problem; indeed, the ribosome is an intricate ribonucleoprotein complex with a multitude of protein constituents present in equimolar amounts. Coordination of the synthesis of these RPs presents a major challenge to the cell and is a result of the sum total of all regulatory mechanisms, i.e., transcriptional, posttranscriptional, translational, posttranslational, on each RP gene. The presence of multiple (often more than two) functional genes encoding each RP substantially make more complex coordinated expression [29]. Chaetognaths, which seem to date unique among animals in carrying multiple paralogous RP functional genes, contradict the current knowledge regarding coordinated systems of RP gene expression in animals. This is probably another prove of the uniqueness of this phylum among animals, as already focused at the anatomical and histological levels. In the future, comparison of chaetognaths versus other animals RP genes regulation will provide fruitful data.

In spite the use of several methods, the phylogenetic relationship of chaetognath is not resolved by the present study. Two biases appear likely to affect our reconstructions, the LBA artefact, and the composition artefact, evidenced by contrasting G+C levels on least constrained third codon positions. Such artefacts may lead to wrong clades with strong branch support and we suspect this is the case for the chaetognath and Drosophila "clade". However, for the second type of bias, the ML non stationary analyses which allow G+C content to vary, still groups the chaetognath with Drosophila, which are long branch species. Furthermore, the second codon position and amino-acid datasets should be much less susceptible to the composition bias but yield the same group. The fact that the non homogeneous amino-acid model CAT, shown to be the most robust method against LBA [64], although at the cost of lower posterior support values [65], yielded a topology that did not join chaetognath and Drosophila suggests that LBA are more important biases than composition artefacts to infer chaetognath phylogenetic relationships. This analysis, which does not group "long branch" species with similar base compositions (chaetognath and Drosophila), is also in agreement with previous works such as Marletaz et al. [20], although the posterior supports are very low, as expected with this method [65]. Therefore, the LBA artefact seems to affect our phylogenetic reconstruction more than the base composition bias, since the methods which are supposed to "correct" for GC-content variation among lineages do not change the topology obtained with more standard methods, while the method supposed to correct for LBA does change it.

Marletaz et al. [20] building a dataset of *S. cephaloptera *RP genes concatenated for 17 taxa, recovered the deuterostomian clade with high bootstrap support, whereas the chaetognaths clustered strongly with protostomes (bootstrap 98%) and their position as a sister group to all other protostomes was supported by weak bootstrap values (51%). As we analysed the paralogy for all the RP gene families and used, after preliminary phylogenetic analyses, only the paralogs with the shorter branches, we hoped to obtain similar topology but with strongly supported nodes, this is not case probably illustrating that the number of taxa plays a significant role and is of major importance when LBA artefacts are into play. Our thorough phylogenetic analyses, using non homogeneous and non stationary models as well as the CAT mixture model for the first time on that data set, helped to identify and correct specific sources of artefactual branch attraction. We can now predict that improvements to infer phylogenetic relationship of the chaetognath phylum will rely on using the PhyloBayes program with the CAT model on a wider taxonomic dataset than the one we used in the present study, such as that of Marletaz et al. [20]. Moreover, versus this last study, we also had the advantages of choosing only the most conserved RP paralogs (by discarding the divergent ones), however, in spite of these various improvements, present results confirm the difficulty of finding the exact phylogenetic relationships of chaetognaths.

## Conclusion

The analysis of chaetognath RP genes has revealed several interesting and original features. However, it has been impossible to relate the presence of two subtypes of mRNA which differ by their 5' UTR region and the great number of paralogs in a coherent molecular evolution pattern. In the future, footprinting and band shift assays will be carried out to investigate the factor(s) which could bind on the 5' UTR region of the RP genes. In addition, using *in situ *hybridization, putative differential tissue expression of paralogous mRNA will be investigated. As our study has shown that the genome of one individual could contain several functional paralogous genes, this suggests that in this taxon, some unknown mechanisms could avoid the deleterious effects of the presence of paralogous ribosomal proteins such as apoptosis or cancer. Probably each paralogous protein has specific functions, one of the paralogs play its role in the ribosome, while the other could have specific extra-ribosomal functions in cells; however, a ribosome heterogeneity where each ribosome is constituted by a class of rRNA associated with a class of RP paralogs could not be excluded, and even the two mechanisms could co-existed.

## Methods

### *Spadella cephaloptera *database of expressed sequence tags

For this study, we have analysed a collection of expressed sequence tags (ESTs) from the chaetognath *S. cephaloptera*, a collection previously assembled by Marletaz et al. [20]. Briefly, cDNA library was made from mRNAs isolated from various embryonic stages of this chaetognath species (from 0 to 48 hours after hatching) using the Lambda-triplex 2 vector. The 5'-ends of 11,254 clones from this library have been sequenced and after annotation analyses, the homology relations have been assigned to 2396 clones corresponding to the transcripts of 792 different genes. Annotated sequences have been submitted by Marletaz et al. [20] on the EMBL website and available online under the accession numbers CR940385 to CR954140.

### Transcription factor binding sites

Potential transcription factor binding sites within the leader mRNA sequences were identified using two prediction programs. The option allowing analysis of orthologous pairs of sequences within ConSite [66] was used to scrutinize numerous aligned chaetognath regions which are homologous in their 5'-ends. All transcription factor binding sites indicated were confirmed against the databases held in ConSite and TFSEARCH [67].

### SSU and LSU ribosomal proteins

Amino acid sequences of RP from rat (*Rattus norvegicus*), fly (*Drosophila melanogaster*), sponge (*Suberites domuncula*) and fungi (*Saccharomyces cerevisiae*) were extracted from corresponding databases. Concerning mammalian, RPs from rat and human are highly conserved and are nearly identical; however, rat, instead of human RPs, were used for comparison, because rat has been the mammalian model organism for the study of RPs for the last 30 years (for review [35]) and human RP sequences are mostly by products of the human genome project. In addition, to date, the complete set of RPs is unknown for any species of molluscs and echinoderms; however, multiple sequences for most RPs belonging to various species are known and we have used RPs from molluscs (mostly two bivalve species: *Crassostrea gigas *and *Argopecten irradians*) and echinoderms (mostly *Strongylocentrotus purpuratus*) that showed highest percentage of aa identity with their homologues from rat. Thus, a total of 38 types of homologous proteins have been obtained, although some of them are partial. EMBL accession numbers of all the complete chaetognath RPs and of the other taxa are in separate tables (Table 1) and [see Additional file 1] respectively.

### Concatenation of ribosomal protein sequences

Sequences of RPs from 6 metazoan species and/or taxa, as well as yeast RPs, were concatenated into 7 respective mega-sequences in the same relative order of proteins. From *S. cephaloptera, R. norvegicus*, echinoderms, molluscs, *D. melanogaster*, *S. domuncula *and *S. cerevisiae*, respectively, 1/the mega-sequences of RPs consisted of 5923, 7175, 6012, 5802, 7291, 6962 and 6748 amino acids; 2/the RPs mega-sequences from SSU comprise 3348, 3534, 3528, 3298, 3515, 3519 and 3419 residues, and the LSU are 2575, 3641, 2484, 2504, 3776, 3443 and 3329 amino acid long.

### Sequence analysis

TBLASTN (Basic Local Alignment Search Tool) was used to identify single ESTs or EST clusters encoding homologues of rat RPs in *S. cephaloptera *database. ESTs encoding RPs were translated and analyzed using Translate and ProtParam tools at ExPASy proteomics server [68]. Chaetognath RPs were further analyzed by NCBI CD search of Conserved Domain Database (CDD) with Reverse Position Specific BLAST. Searches for the occurrence of patterns, profiles and motifs in RPs were performed by Prosite at ExPASy [70]. Multiple alignments of individual RPs or mega-sequences of SSU, LSU and all RPs were obtained using a multiple sequence alignment editor BioEdit version 7 [69]. Statistical data were extracted from GeneDoc [71].

### DNA extraction and amplification of ribosomal genes

Adult specimens of the benthic species *S. cephaloptera *have been caught during spring and summer 2006 in a marine meadows east of Marseilles (Brusc lagoon, France). In the laboratory, samples were kept in aquaria containing natural sea water and placed in a constant temperature at 21 ± 1°C where they were maintained under natural light cycle. DNAs from 3 adult individuals have been extracted separately using CTAB method [72]. Then, ribosomal paralogous genes were amplified by polymerase chain reaction (PCR) using specific primers (Table 4). The 25 μl PCR reaction mix contained 100 ng template DNA, 2.5 μl *Taq *DNA polymerase buffer 10×, 1 μl dNTP mix (50 μM), 1 μl of each primer (20 μM), and 1U *Taq *DNA polymerase (Promega). Samples were amplified during 30 cycles under the following regime: 94°C for 1 min, 45 to 61°C for 30 sec (according the couple of primers), and 72°C for 30 sec. The forward primer sequences for genes bearing the putative TTT and TAC sites are respectively TTT: 5'-GGAAGCTTATAATTGAGTAGTTT-3' and TAC: 5'-GGAAGCTTATTATTAAGTACTAC-3'. The sequences of the reverse primers were: S8-1R: 5'-CCGCTTCGCTCAATTTGGCGC-3', S8-2R: 5'-CGGCTTCGCTCAATTTAGAAC-3', S8-3R: 5'-CGGCTTCACTCAGTTTGGTTG-3', S25-1R: 5'-CGGCATCTTCAAATTCCGAAACG-3', S25-2R: 5'-CTCAAGCTTGGACGAGTAAG-3', S25-3R: 5'-CGCCGGCCGCCTTGGGTGGC-3', L15-1R: 5'-CCCATAGTGATAATCCTGACC-3', L15-2R: 5'-ACACGGGTGAAGCCCCCC-3', L27a-1R: 5'-GCTGTCCTTTCGACATCTTTAC-3', L27a-2R: 5'-GGGCTTCAACTTGGACATG-3'.

### Phylogenetic analyses

In each *S. cephaloptera *RP family, in order to to minimize the artifact of LBA, when several paralogs have been found, only the sequences giving the shortest branch in phylogenetic analyses were chosen; these paralogs which have been named isoform 1 are also the closest to the animal consensus sequences.

Firstly, using seven taxa dataset, RP DNA sequences were translated into amino acid sequences to overcome the problem of GC content differences [73]. Phylogenetic trees based on amino acid sequences were constructed by four methods, all from the Phylip package: Prodist [using two distance methods: Fitch and Neighbor-joining (NJ)], Protpars [maximum parsimony (MP)], Proml [maximum likelihood (ML)] [74]; all parameters were set to default values. Bootstrap analysis on 1000 replicates was performed with Seqboot from the same software package. This dataset contained 5412 amino acids, with a limited number of missing data. For tree construction, positions with gaps in one or more sequences were excluded.

In a second approach, we restricted the analysis to animals, to limit the biases generated by too divergent outgroups, and eliminating all the sites containing missing data. The addition of more divergent outgroups to a given dataset already containing an outgroup is known to decrease the reliability of phylogenetic reconstruction, rather than increasing it. This is due to a phenomenon analogous to the LBA artefact. Therefore, since the outgroup status of sponges is not dubious (and confirmed by our 7 species dataset analyses) it is better not to use the yeast in more refined analyses. This resulted in a 4638 amino-acid dataset for 6 species with sponges as the outgroup. This dataset was analysed by a range of methods including the most recent ones; they are presented below, with their relative advantages and susceptibility to artefacts, and either using the amino-acid or the nucleotide sequence data, and in the last case, either using codon models, or nucleotide models with different partitions of codon positions. From that combination of analyses we will identify which are the most important artefacts and which are the solutions.

We first used a homogeneous stationary Maximum likelihood method implemented by PhyML [75]. On the amino-acid data set, we used the WAG empirical model of evolution, estimating the proportion of invariant sites by maximising the likelihood, and assuming a gamma shape distribution of rates of substitution among sites with a value of 2 for the gamma parameter. The corresponding 13914 nucleotide sequence alignments were also analysed with PhyML. We used several codon position partitions (first and second positions, second position only, and all three positions) for most types of analyses. MrAIC [76] was used to determine the best model of nucleotide evolution using Akaike criteria. The best model selected was then used to obtain a consensus tree from 100 bootstraps of PhyML analyses, and we computed approximate likelihood ratio test branch supports for this model using aLRT-PhyML [77]. PhyML uses a starting tree to explore the space of possible trees: we used both the BIONJ starting tree option (in which the starting tree is inferred by BIONJ) and a "user defined starting tree", where the chaetognath was at the base of a protostome clade, to possibly avoid a bias of LBA at this step since BIONJ is sensible to LBA (see results and discussion). ML trees were also reconstructed assuming codon models of substitution, which combine the information at the amino-acid level (non synonymous changes, less saturation) and nucleotide level (compositional biases, codon preference) using the HyPHY package [78].

Non ML methods were also used on these data sets using MEGA version 3.1 [79], parsimony, and Neighbour Joining with the LogDet distance, in an attempt to overcome the problem of mutual attraction of the branches which have the same compositional bias. In effect, even the most rich parameter models of classical available ML methods are stationary and homogeneous (i.e., they assume that the same matrix of substitution applies to all branches of the tree and all sites, therefore compositional biases may lead to wrong topology with high bootstrap and high aLRT branch support). Using MEGA, we computed the base composition of each taxon, and performed relative tests of evolutionary rate among taxa, allowing to identify taxa susceptible to be artefactually joined because of similar nucleotide composition or evolutionary rates (long branch attraction).

A non stationary ML method allowing to to relax the assumption of identical substitution processes among branches was used: This method takes into account variable G+C levels [80] and was implemented using the program nhPhyml [81] with three starting trees corresponding to the three possible topologies obtained within the Protostoma + chaetognath group, assuming Deuterostoma (rat and echinoderm sequences) form a distinct clade, considering the second position of the codons only, or the first and second positions.

Bayesian phylogenetic analyses of the amino acid dataset were performed using softwares MrBayes version 3.1.1 [82] and PhyloBayes version 2.1 [65]. We used the GTR (General Time Reversible) model of MrBayes, chains were run for 1,000,000 cycle long, saving a sample each 100 cycles. A burnin period of 2,000 samples was discarded and the remaining 8,000 samples were used for the consensus topology computation. We moreover used the mixture model CAT implemented in PhyloBayes, which allows a mixture of processes of substitution to be distributed across sites and does not make the assumption of the same amino-acid substitution process across sites. This model has been shown to be very efficient against LBA artefacts [65]. Chains were run for a default length of 1100 cycles, each cycle yielding a sufficiently decorrelated sample. The first 100 samples were discarded as burnin and the consensus topology was extract from the 1000 remaining samples. Both experiments, under GTR and CAT, used four discrete categories of rates across sites and were run twice in order to check chains' convergence.

## Authors' contributions

RMB and JPC have caught *S. cephaloptera *individuals, kept them in aquaria, prepared samples, assisted in data analyses and revised critically the manuscript. AC and SB perfected phylogenetic analyses and revised critically the manuscript. EF conceived of the study, designed and perfected the PCRs and data analyses, and wrote the manuscript. All authors read and approved the final manuscript.

## Supplementary Material

Additional file 1 List of the animal and fungi ribosomal proteins used in the phylogenetical analyses. Click here for file
