# Supplementary material for: Translational machinery of the chaetognath Spadella cephaloptera: a transcriptomic approach to the analysis of cytosolic ribosomal protein genes and their expression
Source: BMC Evol Biol. 2007 Aug 28;7:146. doi: 10.1186/1471-2148-7-146 (PMC2020476; doi:10.1186/1471-2148-7-146)
Supplement: Additional file 1 — List of the animal and fungi ribosomal proteins used in the phylogenetical analyses. [file 1471-2148-7-146-S1.doc]

**List of the animal and fungi ribosomal proteins used in the phylogenetical analyses.**

| Protein name | Acc. n° mollusk | Acc. n° echinoderm | Acc. n° sponge:  *Suberites domuncula* | Acc. n° fly : *Drosophila melanogaster* | Acc. n° rat: *Rattus norvegicus* | Acc. n° yeast: *Saccharomyces cerevisiae* |
| --- | --- | --- | --- | --- | --- | --- |
| *SSU ribosomal proteins* | | | | | | |
| S2 | GenBank:AAM94271 | RefSeq:XP_781777 | GenBank:AAX48877 | PIR:[S30395](http://www.sciencedirect.com/science?_ob=RedirectURL&_method=externObjLink&_locator=genbank&_cdi=4941&_plusSign=%2B&_targetURL=http%253A%252F%252Fwww.ncbi.nlm.nih.gov%252Fentrez%252Fquery.fcgi%253Fcmd%253Dsearch%2526db%253Dnucleotide%2526doptcmdl%253Dgenbank%2526term%253DS30395%5Baccn%5D) | RefSeq:[NP_114026](http://www.sciencedirect.com/science?_ob=RedirectURL&_method=externObjLink&_locator=genbank&_cdi=4941&_plusSign=%2B&_targetURL=http%253A%252F%252Fwww.ncbi.nlm.nih.gov%252Fentrez%252Fquery.fcgi%253Fcmd%253Dsearch%2526db%253Dnucleotide%2526doptcmdl%253Dgenbank%2526term%253DNP_114026%5Baccn%5D) | GenBank:[AAA63576](http://www.sciencedirect.com/science?_ob=RedirectURL&_method=externObjLink&_locator=genbank&_cdi=4941&_plusSign=%2B&_targetURL=http%253A%252F%252Fwww.ncbi.nlm.nih.gov%252Fentrez%252Fquery.fcgi%253Fcmd%253Dsearch%2526db%253Dnucleotide%2526doptcmdl%253Dgenbank%2526term%253DAAA63576%5Baccn%5D) |
| S3 | EMBL:CAD91437 | RefSeq:XP_798304 | GenBank:AAX48878 | EMBL:[CAA51425](http://www.sciencedirect.com/science?_ob=RedirectURL&_method=externObjLink&_locator=genbank&_cdi=4941&_plusSign=%2B&_targetURL=http%253A%252F%252Fwww.ncbi.nlm.nih.gov%252Fentrez%252Fquery.fcgi%253Fcmd%253Dsearch%2526db%253Dnucleotide%2526doptcmdl%253Dgenbank%2526term%253DCAA51425%5Baccn%5D) | RefSeq:[NP_001009239](http://www.sciencedirect.com/science?_ob=RedirectURL&_method=externObjLink&_locator=genbank&_cdi=4941&_plusSign=%2B&_targetURL=http%253A%252F%252Fwww.ncbi.nlm.nih.gov%252Fentrez%252Fquery.fcgi%253Fcmd%253Dsearch%2526db%253Dnucleotide%2526doptcmdl%253Dgenbank%2526term%253DNP_001009239%5Baccn%5D) | Swiss-Prot:[P05750](http://www.sciencedirect.com/science?_ob=RedirectURL&_method=externObjLink&_locator=genbank&_cdi=4941&_plusSign=%2B&_targetURL=http%253A%252F%252Fwww.ncbi.nlm.nih.gov%252Fentrez%252Fquery.fcgi%253Fcmd%253Dsearch%2526db%253Dnucleotide%2526doptcmdl%253Dgenbank%2526term%253DP05750%5Baccn%5D) |
| S3a | EMBL:CAA48558 | RefSeq:XP_780038 | GenBank:AAX48879 | Swiss-Prot:[P55830](http://www.sciencedirect.com/science?_ob=RedirectURL&_method=externObjLink&_locator=genbank&_cdi=4941&_plusSign=%2B&_targetURL=http%253A%252F%252Fwww.ncbi.nlm.nih.gov%252Fentrez%252Fquery.fcgi%253Fcmd%253Dsearch%2526db%253Dnucleotide%2526doptcmdl%253Dgenbank%2526term%253DP55830%5Baccn%5D) | RefSeq:[NP_058849](http://www.sciencedirect.com/science?_ob=RedirectURL&_method=externObjLink&_locator=genbank&_cdi=4941&_plusSign=%2B&_targetURL=http%253A%252F%252Fwww.ncbi.nlm.nih.gov%252Fentrez%252Fquery.fcgi%253Fcmd%253Dsearch%2526db%253Dnucleotide%2526doptcmdl%253Dgenbank%2526term%253DNP_058849%5Baccn%5D) | RefSeq:[NP_013546](http://www.sciencedirect.com/science?_ob=RedirectURL&_method=externObjLink&_locator=genbank&_cdi=4941&_plusSign=%2B&_targetURL=http%253A%252F%252Fwww.ncbi.nlm.nih.gov%252Fentrez%252Fquery.fcgi%253Fcmd%253Dsearch%2526db%253Dnucleotide%2526doptcmdl%253Dgenbank%2526term%253DNP_013546%5Baccn%5D) |
| S4 | GenBank:AAN05593 | RefSeq:XP_780509 | GenBank:AAX48880 | Swiss-Prot:[P41042](http://www.sciencedirect.com/science?_ob=RedirectURL&_method=externObjLink&_locator=genbank&_cdi=4941&_plusSign=%2B&_targetURL=http%253A%252F%252Fwww.ncbi.nlm.nih.gov%252Fentrez%252Fquery.fcgi%253Fcmd%253Dsearch%2526db%253Dnucleotide%2526doptcmdl%253Dgenbank%2526term%253DP41042%5Baccn%5D) | Swiss-Prot:[P62703](http://www.sciencedirect.com/science?_ob=RedirectURL&_method=externObjLink&_locator=genbank&_cdi=4941&_plusSign=%2B&_targetURL=http%253A%252F%252Fwww.ncbi.nlm.nih.gov%252Fentrez%252Fquery.fcgi%253Fcmd%253Dsearch%2526db%253Dnucleotide%2526doptcmdl%253Dgenbank%2526term%253DP62703%5Baccn%5D) | Swiss-Prot:[P05753](http://www.sciencedirect.com/science?_ob=RedirectURL&_method=externObjLink&_locator=genbank&_cdi=4941&_plusSign=%2B&_targetURL=http%253A%252F%252Fwww.ncbi.nlm.nih.gov%252Fentrez%252Fquery.fcgi%253Fcmd%253Dsearch%2526db%253Dnucleotide%2526doptcmdl%253Dgenbank%2526term%253DP05753%5Baccn%5D) |
| S6 | GenBank:AAG60623 | EMBL:CAD27733 | GenBank:AAX48882 | Swiss-Prot:[P29327](http://www.sciencedirect.com/science?_ob=RedirectURL&_method=externObjLink&_locator=genbank&_cdi=4941&_plusSign=%2B&_targetURL=http%253A%252F%252Fwww.ncbi.nlm.nih.gov%252Fentrez%252Fquery.fcgi%253Fcmd%253Dsearch%2526db%253Dnucleotide%2526doptcmdl%253Dgenbank%2526term%253DP29327%5Baccn%5D) | RefSeq:[NP_058856](http://www.sciencedirect.com/science?_ob=RedirectURL&_method=externObjLink&_locator=genbank&_cdi=4941&_plusSign=%2B&_targetURL=http%253A%252F%252Fwww.ncbi.nlm.nih.gov%252Fentrez%252Fquery.fcgi%253Fcmd%253Dsearch%2526db%253Dnucleotide%2526doptcmdl%253Dgenbank%2526term%253DNP_058856%5Baccn%5D) | Swiss-Prot:[P02365](http://www.sciencedirect.com/science?_ob=RedirectURL&_method=externObjLink&_locator=genbank&_cdi=4941&_plusSign=%2B&_targetURL=http%253A%252F%252Fwww.ncbi.nlm.nih.gov%252Fentrez%252Fquery.fcgi%253Fcmd%253Dsearch%2526db%253Dnucleotide%2526doptcmdl%253Dgenbank%2526term%253DP02365%5Baccn%5D) |
| S7 | GenBank:AAN05602 | RefSeq:XP_789754 | GenBank:AAX48883 | RefSeq:[NP_651782](http://www.sciencedirect.com/science?_ob=RedirectURL&_method=externObjLink&_locator=genbank&_cdi=4941&_plusSign=%2B&_targetURL=http%253A%252F%252Fwww.ncbi.nlm.nih.gov%252Fentrez%252Fquery.fcgi%253Fcmd%253Dsearch%2526db%253Dnucleotide%2526doptcmdl%253Dgenbank%2526term%253DNP_651782%5Baccn%5D) | Swiss-Prot:[P62083](http://www.sciencedirect.com/science?_ob=RedirectURL&_method=externObjLink&_locator=genbank&_cdi=4941&_plusSign=%2B&_targetURL=http%253A%252F%252Fwww.ncbi.nlm.nih.gov%252Fentrez%252Fquery.fcgi%253Fcmd%253Dsearch%2526db%253Dnucleotide%2526doptcmdl%253Dgenbank%2526term%253DP62083%5Baccn%5D) | Swiss-Prot:[P26786](http://www.sciencedirect.com/science?_ob=RedirectURL&_method=externObjLink&_locator=genbank&_cdi=4941&_plusSign=%2B&_targetURL=http%253A%252F%252Fwww.ncbi.nlm.nih.gov%252Fentrez%252Fquery.fcgi%253Fcmd%253Dsearch%2526db%253Dnucleotide%2526doptcmdl%253Dgenbank%2526term%253DP26786%5Baccn%5D) |
| S8 | EMBL:CAD91426 | RefSeq:XP_780905 | GenBank:AAX48884 | GenBank:[AAM48453](http://www.sciencedirect.com/science?_ob=RedirectURL&_method=externObjLink&_locator=genbank&_cdi=4941&_plusSign=%2B&_targetURL=http%253A%252F%252Fwww.ncbi.nlm.nih.gov%252Fentrez%252Fquery.fcgi%253Fcmd%253Dsearch%2526db%253Dnucleotide%2526doptcmdl%253Dgenbank%2526term%253DAAM48453%5Baccn%5D) | RefSeq:[NP_113894](http://www.sciencedirect.com/science?_ob=RedirectURL&_method=externObjLink&_locator=genbank&_cdi=4941&_plusSign=%2B&_targetURL=http%253A%252F%252Fwww.ncbi.nlm.nih.gov%252Fentrez%252Fquery.fcgi%253Fcmd%253Dsearch%2526db%253Dnucleotide%2526doptcmdl%253Dgenbank%2526term%253DNP_113894%5Baccn%5D) | EMBL:[CAA81525](http://www.sciencedirect.com/science?_ob=RedirectURL&_method=externObjLink&_locator=genbank&_cdi=4941&_plusSign=%2B&_targetURL=http%253A%252F%252Fwww.ncbi.nlm.nih.gov%252Fentrez%252Fquery.fcgi%253Fcmd%253Dsearch%2526db%253Dnucleotide%2526doptcmdl%253Dgenbank%2526term%253DCAA81525%5Baccn%5D) |
| S9 | GenBank:AAT76788 | RefSeq:XP_791000 | GenBank:AAX48885 | Swiss-Prot:[P55935](http://www.sciencedirect.com/science?_ob=RedirectURL&_method=externObjLink&_locator=genbank&_cdi=4941&_plusSign=%2B&_targetURL=http%253A%252F%252Fwww.ncbi.nlm.nih.gov%252Fentrez%252Fquery.fcgi%253Fcmd%253Dsearch%2526db%253Dnucleotide%2526doptcmdl%253Dgenbank%2526term%253DP55935%5Baccn%5D) | RefSeq:[NP_112370](http://www.sciencedirect.com/science?_ob=RedirectURL&_method=externObjLink&_locator=genbank&_cdi=4941&_plusSign=%2B&_targetURL=http%253A%252F%252Fwww.ncbi.nlm.nih.gov%252Fentrez%252Fquery.fcgi%253Fcmd%253Dsearch%2526db%253Dnucleotide%2526doptcmdl%253Dgenbank%2526term%253DNP_112370%5Baccn%5D) | Swiss-Prot:[O13516](http://www.sciencedirect.com/science?_ob=RedirectURL&_method=externObjLink&_locator=genbank&_cdi=4941&_plusSign=%2B&_targetURL=http%253A%252F%252Fwww.ncbi.nlm.nih.gov%252Fentrez%252Fquery.fcgi%253Fcmd%253Dsearch%2526db%253Dnucleotide%2526doptcmdl%253Dgenbank%2526term%253DO13516%5Baccn%5D) |
| S11 | EMBL:CAD91419 | RefSeq:XP_784254 | GenBank:AAX48887 | RefSeq:[NP_610747](http://www.sciencedirect.com/science?_ob=RedirectURL&_method=externObjLink&_locator=genbank&_cdi=4941&_plusSign=%2B&_targetURL=http%253A%252F%252Fwww.ncbi.nlm.nih.gov%252Fentrez%252Fquery.fcgi%253Fcmd%253Dsearch%2526db%253Dnucleotide%2526doptcmdl%253Dgenbank%2526term%253DNP_610747%5Baccn%5D) | RefSeq:[NP_112372](http://www.sciencedirect.com/science?_ob=RedirectURL&_method=externObjLink&_locator=genbank&_cdi=4941&_plusSign=%2B&_targetURL=http%253A%252F%252Fwww.ncbi.nlm.nih.gov%252Fentrez%252Fquery.fcgi%253Fcmd%253Dsearch%2526db%253Dnucleotide%2526doptcmdl%253Dgenbank%2526term%253DNP_112372%5Baccn%5D) | EMBL:[CAA87804](http://www.sciencedirect.com/science?_ob=RedirectURL&_method=externObjLink&_locator=genbank&_cdi=4941&_plusSign=%2B&_targetURL=http%253A%252F%252Fwww.ncbi.nlm.nih.gov%252Fentrez%252Fquery.fcgi%253Fcmd%253Dsearch%2526db%253Dnucleotide%2526doptcmdl%253Dgenbank%2526term%253DCAA87804%5Baccn%5D) |
| S13 | GenBank:AAN05601 | RefSeq:XP_786303 | GenBank:AAX48889 | RefSeq:[NP_476938](http://www.sciencedirect.com/science?_ob=RedirectURL&_method=externObjLink&_locator=genbank&_cdi=4941&_plusSign=%2B&_targetURL=http%253A%252F%252Fwww.ncbi.nlm.nih.gov%252Fentrez%252Fquery.fcgi%253Fcmd%253Dsearch%2526db%253Dnucleotide%2526doptcmdl%253Dgenbank%2526term%253DNP_476938%5Baccn%5D) | RefSeq:[NP_569116](http://www.sciencedirect.com/science?_ob=RedirectURL&_method=externObjLink&_locator=genbank&_cdi=4941&_plusSign=%2B&_targetURL=http%253A%252F%252Fwww.ncbi.nlm.nih.gov%252Fentrez%252Fquery.fcgi%253Fcmd%253Dsearch%2526db%253Dnucleotide%2526doptcmdl%253Dgenbank%2526term%253DNP_569116%5Baccn%5D) | Swiss-Prot:[P05756](http://www.sciencedirect.com/science?_ob=RedirectURL&_method=externObjLink&_locator=genbank&_cdi=4941&_plusSign=%2B&_targetURL=http%253A%252F%252Fwww.ncbi.nlm.nih.gov%252Fentrez%252Fquery.fcgi%253Fcmd%253Dsearch%2526db%253Dnucleotide%2526doptcmdl%253Dgenbank%2526term%253DP05756%5Baccn%5D) |
| S14 | GenBank:AAO11522 | RefSeq:XP_780390 | GenBank:AAX48890 | Swiss-Prot:[P14130](http://www.sciencedirect.com/science?_ob=RedirectURL&_method=externObjLink&_locator=genbank&_cdi=4941&_plusSign=%2B&_targetURL=http%253A%252F%252Fwww.ncbi.nlm.nih.gov%252Fentrez%252Fquery.fcgi%253Fcmd%253Dsearch%2526db%253Dnucleotide%2526doptcmdl%253Dgenbank%2526term%253DP14130%5Baccn%5D) | RefSeq:[NP_073163](http://www.sciencedirect.com/science?_ob=RedirectURL&_method=externObjLink&_locator=genbank&_cdi=4941&_plusSign=%2B&_targetURL=http%253A%252F%252Fwww.ncbi.nlm.nih.gov%252Fentrez%252Fquery.fcgi%253Fcmd%253Dsearch%2526db%253Dnucleotide%2526doptcmdl%253Dgenbank%2526term%253DNP_073163%5Baccn%5D) | Swiss-Prot:[P39516](http://www.sciencedirect.com/science?_ob=RedirectURL&_method=externObjLink&_locator=genbank&_cdi=4941&_plusSign=%2B&_targetURL=http%253A%252F%252Fwww.ncbi.nlm.nih.gov%252Fentrez%252Fquery.fcgi%253Fcmd%253Dsearch%2526db%253Dnucleotide%2526doptcmdl%253Dgenbank%2526term%253DP39516%5Baccn%5D) |
| S15 | GenBank:AAN05605 | RefSeq:XP_780510 | GenBank:AAX48891 | GenBank:[AAF57984](http://www.sciencedirect.com/science?_ob=RedirectURL&_method=externObjLink&_locator=genbank&_cdi=4941&_plusSign=%2B&_targetURL=http%253A%252F%252Fwww.ncbi.nlm.nih.gov%252Fentrez%252Fquery.fcgi%253Fcmd%253Dsearch%2526db%253Dnucleotide%2526doptcmdl%253Dgenbank%2526term%253DAAF57984%5Baccn%5D) | RefSeq:[NP_058847](http://www.sciencedirect.com/science?_ob=RedirectURL&_method=externObjLink&_locator=genbank&_cdi=4941&_plusSign=%2B&_targetURL=http%253A%252F%252Fwww.ncbi.nlm.nih.gov%252Fentrez%252Fquery.fcgi%253Fcmd%253Dsearch%2526db%253Dnucleotide%2526doptcmdl%253Dgenbank%2526term%253DNP_058847%5Baccn%5D) | Swiss-Prot:[Q01855](http://www.sciencedirect.com/science?_ob=RedirectURL&_method=externObjLink&_locator=genbank&_cdi=4941&_plusSign=%2B&_targetURL=http%253A%252F%252Fwww.ncbi.nlm.nih.gov%252Fentrez%252Fquery.fcgi%253Fcmd%253Dsearch%2526db%253Dnucleotide%2526doptcmdl%253Dgenbank%2526term%253DQ01855%5Baccn%5D) |
| S16 | GenBank:AAM09676 | RefSeq:XP_780037 | GenBank:AAX48893 | RefSeq:[NP_611685](http://www.sciencedirect.com/science?_ob=RedirectURL&_method=externObjLink&_locator=genbank&_cdi=4941&_plusSign=%2B&_targetURL=http%253A%252F%252Fwww.ncbi.nlm.nih.gov%252Fentrez%252Fquery.fcgi%253Fcmd%253Dsearch%2526db%253Dnucleotide%2526doptcmdl%253Dgenbank%2526term%253DNP_611685%5Baccn%5D) | Swiss-Prot:[P62250](http://www.sciencedirect.com/science?_ob=RedirectURL&_method=externObjLink&_locator=genbank&_cdi=4941&_plusSign=%2B&_targetURL=http%253A%252F%252Fwww.ncbi.nlm.nih.gov%252Fentrez%252Fquery.fcgi%253Fcmd%253Dsearch%2526db%253Dnucleotide%2526doptcmdl%253Dgenbank%2526term%253DP62250%5Baccn%5D) | Swiss-Prot:[P40213](http://www.sciencedirect.com/science?_ob=RedirectURL&_method=externObjLink&_locator=genbank&_cdi=4941&_plusSign=%2B&_targetURL=http%253A%252F%252Fwww.ncbi.nlm.nih.gov%252Fentrez%252Fquery.fcgi%253Fcmd%253Dsearch%2526db%253Dnucleotide%2526doptcmdl%253Dgenbank%2526term%253DP40213%5Baccn%5D) |
| S17 | GenBank:AAN05594 | RefSeq:XP_783043 | GenBank:AAX48894 | Swiss-Prot:[P17704](http://www.sciencedirect.com/science?_ob=RedirectURL&_method=externObjLink&_locator=genbank&_cdi=4941&_plusSign=%2B&_targetURL=http%253A%252F%252Fwww.ncbi.nlm.nih.gov%252Fentrez%252Fquery.fcgi%253Fcmd%253Dsearch%2526db%253Dnucleotide%2526doptcmdl%253Dgenbank%2526term%253DP17704%5Baccn%5D) | Swiss-Prot:[P04644](http://www.sciencedirect.com/science?_ob=RedirectURL&_method=externObjLink&_locator=genbank&_cdi=4941&_plusSign=%2B&_targetURL=http%253A%252F%252Fwww.ncbi.nlm.nih.gov%252Fentrez%252Fquery.fcgi%253Fcmd%253Dsearch%2526db%253Dnucleotide%2526doptcmdl%253Dgenbank%2526term%253DP04644%5Baccn%5D) | Swiss-Prot:[P02407](http://www.sciencedirect.com/science?_ob=RedirectURL&_method=externObjLink&_locator=genbank&_cdi=4941&_plusSign=%2B&_targetURL=http%253A%252F%252Fwww.ncbi.nlm.nih.gov%252Fentrez%252Fquery.fcgi%253Fcmd%253Dsearch%2526db%253Dnucleotide%2526doptcmdl%253Dgenbank%2526term%253DP02407%5Baccn%5D) |
| S18 | DDBJ:BAE16014 | RefSeq:XP_784070 | GenBank:AAX48895 | RefSeq:[NP_476964](http://www.sciencedirect.com/science?_ob=RedirectURL&_method=externObjLink&_locator=genbank&_cdi=4941&_plusSign=%2B&_targetURL=http%253A%252F%252Fwww.ncbi.nlm.nih.gov%252Fentrez%252Fquery.fcgi%253Fcmd%253Dsearch%2526db%253Dnucleotide%2526doptcmdl%253Dgenbank%2526term%253DNP_476964%5Baccn%5D) | RefSeq:[NP_998722](http://www.sciencedirect.com/science?_ob=RedirectURL&_method=externObjLink&_locator=genbank&_cdi=4941&_plusSign=%2B&_targetURL=http%253A%252F%252Fwww.ncbi.nlm.nih.gov%252Fentrez%252Fquery.fcgi%253Fcmd%253Dsearch%2526db%253Dnucleotide%2526doptcmdl%253Dgenbank%2526term%253DNP_998722%5Baccn%5D) | Swiss-Prot:[P35271](http://www.sciencedirect.com/science?_ob=RedirectURL&_method=externObjLink&_locator=genbank&_cdi=4941&_plusSign=%2B&_targetURL=http%253A%252F%252Fwww.ncbi.nlm.nih.gov%252Fentrez%252Fquery.fcgi%253Fcmd%253Dsearch%2526db%253Dnucleotide%2526doptcmdl%253Dgenbank%2526term%253DP35271%5Baccn%5D) |
| S19 | GenBank:AAB09536 | RefSeq:XP_783440 | GenBank:AAX48896 | Swiss-Prot:[P39018](http://www.sciencedirect.com/science?_ob=RedirectURL&_method=externObjLink&_locator=genbank&_cdi=4941&_plusSign=%2B&_targetURL=http%253A%252F%252Fwww.ncbi.nlm.nih.gov%252Fentrez%252Fquery.fcgi%253Fcmd%253Dsearch%2526db%253Dnucleotide%2526doptcmdl%253Dgenbank%2526term%253DP39018%5Baccn%5D) | Swiss-Prot:[P17074](http://www.sciencedirect.com/science?_ob=RedirectURL&_method=externObjLink&_locator=genbank&_cdi=4941&_plusSign=%2B&_targetURL=http%253A%252F%252Fwww.ncbi.nlm.nih.gov%252Fentrez%252Fquery.fcgi%253Fcmd%253Dsearch%2526db%253Dnucleotide%2526doptcmdl%253Dgenbank%2526term%253DP17074%5Baccn%5D) | Swiss-Prot:[P07280](http://www.sciencedirect.com/science?_ob=RedirectURL&_method=externObjLink&_locator=genbank&_cdi=4941&_plusSign=%2B&_targetURL=http%253A%252F%252Fwww.ncbi.nlm.nih.gov%252Fentrez%252Fquery.fcgi%253Fcmd%253Dsearch%2526db%253Dnucleotide%2526doptcmdl%253Dgenbank%2526term%253DP07280%5Baccn%5D) |
| S20 | EMBL:CAD91428 | RefSeq:XP_782034 | GenBank:AAX48897 | Swiss-Prot:[P55828](http://www.sciencedirect.com/science?_ob=RedirectURL&_method=externObjLink&_locator=genbank&_cdi=4941&_plusSign=%2B&_targetURL=http%253A%252F%252Fwww.ncbi.nlm.nih.gov%252Fentrez%252Fquery.fcgi%253Fcmd%253Dsearch%2526db%253Dnucleotide%2526doptcmdl%253Dgenbank%2526term%253DP55828%5Baccn%5D) | RefSeq:[NP_001007604](http://www.sciencedirect.com/science?_ob=RedirectURL&_method=externObjLink&_locator=genbank&_cdi=4941&_plusSign=%2B&_targetURL=http%253A%252F%252Fwww.ncbi.nlm.nih.gov%252Fentrez%252Fquery.fcgi%253Fcmd%253Dsearch%2526db%253Dnucleotide%2526doptcmdl%253Dgenbank%2526term%253DNP_001007604%5Baccn%5D) | Swiss-Prot:[P38701](http://www.sciencedirect.com/science?_ob=RedirectURL&_method=externObjLink&_locator=genbank&_cdi=4941&_plusSign=%2B&_targetURL=http%253A%252F%252Fwww.ncbi.nlm.nih.gov%252Fentrez%252Fquery.fcgi%253Fcmd%253Dsearch%2526db%253Dnucleotide%2526doptcmdl%253Dgenbank%2526term%253DP38701%5Baccn%5D) |
| S25 | EMBL:CAD91125 | RefSeq:XP_794681 | GenBank:AAX4890 | Swiss-Prot:[P48588](http://www.sciencedirect.com/science?_ob=RedirectURL&_method=externObjLink&_locator=genbank&_cdi=4941&_plusSign=%2B&_targetURL=http%253A%252F%252Fwww.ncbi.nlm.nih.gov%252Fentrez%252Fquery.fcgi%253Fcmd%253Dsearch%2526db%253Dnucleotide%2526doptcmdl%253Dgenbank%2526term%253DP48588%5Baccn%5D) | RefSeq:[NP_001005528](http://www.sciencedirect.com/science?_ob=RedirectURL&_method=externObjLink&_locator=genbank&_cdi=4941&_plusSign=%2B&_targetURL=http%253A%252F%252Fwww.ncbi.nlm.nih.gov%252Fentrez%252Fquery.fcgi%253Fcmd%253Dsearch%2526db%253Dnucleotide%2526doptcmdl%253Dgenbank%2526term%253DNP_001005528%5Baccn%5D) | Swiss-Prot:[P07282](http://www.sciencedirect.com/science?_ob=RedirectURL&_method=externObjLink&_locator=genbank&_cdi=4941&_plusSign=%2B&_targetURL=http%253A%252F%252Fwww.ncbi.nlm.nih.gov%252Fentrez%252Fquery.fcgi%253Fcmd%253Dsearch%2526db%253Dnucleotide%2526doptcmdl%253Dgenbank%2526term%253DP07282%5Baccn%5D) |
| S26 | EMBL:CAB57819 | RefSeq:XP_782030 | GenBank:AAX48902 | Swiss-Prot:[P13008](http://www.sciencedirect.com/science?_ob=RedirectURL&_method=externObjLink&_locator=genbank&_cdi=4941&_plusSign=%2B&_targetURL=http%253A%252F%252Fwww.ncbi.nlm.nih.gov%252Fentrez%252Fquery.fcgi%253Fcmd%253Dsearch%2526db%253Dnucleotide%2526doptcmdl%253Dgenbank%2526term%253DP13008%5Baccn%5D) | RefSeq:[NP_037356](http://www.sciencedirect.com/science?_ob=RedirectURL&_method=externObjLink&_locator=genbank&_cdi=4941&_plusSign=%2B&_targetURL=http%253A%252F%252Fwww.ncbi.nlm.nih.gov%252Fentrez%252Fquery.fcgi%253Fcmd%253Dsearch%2526db%253Dnucleotide%2526doptcmdl%253Dgenbank%2526term%253DNP_037356%5Baccn%5D) | Swiss-Prot:[P39938](http://www.sciencedirect.com/science?_ob=RedirectURL&_method=externObjLink&_locator=genbank&_cdi=4941&_plusSign=%2B&_targetURL=http%253A%252F%252Fwww.ncbi.nlm.nih.gov%252Fentrez%252Fquery.fcgi%253Fcmd%253Dsearch%2526db%253Dnucleotide%2526doptcmdl%253Dgenbank%2526term%253DP39938%5Baccn%5D) |
| S27 | DDBJ:BAE16015 | RefSeq:XP_784036 | GenBank:AAX48903 | RefSeq:[NP_651359](http://www.sciencedirect.com/science?_ob=RedirectURL&_method=externObjLink&_locator=genbank&_cdi=4941&_plusSign=%2B&_targetURL=http%253A%252F%252Fwww.ncbi.nlm.nih.gov%252Fentrez%252Fquery.fcgi%253Fcmd%253Dsearch%2526db%253Dnucleotide%2526doptcmdl%253Dgenbank%2526term%253DNP_651359%5Baccn%5D) | RefSeq:[NP_446049](http://www.sciencedirect.com/science?_ob=RedirectURL&_method=externObjLink&_locator=genbank&_cdi=4941&_plusSign=%2B&_targetURL=http%253A%252F%252Fwww.ncbi.nlm.nih.gov%252Fentrez%252Fquery.fcgi%253Fcmd%253Dsearch%2526db%253Dnucleotide%2526doptcmdl%253Dgenbank%2526term%253DNP_446049%5Baccn%5D) | Swiss-Prot:[P38711](http://www.sciencedirect.com/science?_ob=RedirectURL&_method=externObjLink&_locator=genbank&_cdi=4941&_plusSign=%2B&_targetURL=http%253A%252F%252Fwww.ncbi.nlm.nih.gov%252Fentrez%252Fquery.fcgi%253Fcmd%253Dsearch%2526db%253Dnucleotide%2526doptcmdl%253Dgenbank%2526term%253DP38711%5Baccn%5D) |
| *LSU ribosomal proteins* | | | | | | |
| P1 | GenBank:AAZ39530 | RefSeq:XP_793925 | GenBank:AAX48830 | PIR:[R5FF2E](http://www.sciencedirect.com/science?_ob=RedirectURL&_method=externObjLink&_locator=genbank&_cdi=4941&_plusSign=%2B&_targetURL=http%253A%252F%252Fwww.ncbi.nlm.nih.gov%252Fentrez%252Fquery.fcgi%253Fcmd%253Dsearch%2526db%253Dnucleotide%2526doptcmdl%253Dgenbank%2526term%253DR5FF2E%5Baccn%5D) | RefSeq:[NP_001007605](http://www.sciencedirect.com/science?_ob=RedirectURL&_method=externObjLink&_locator=genbank&_cdi=4941&_plusSign=%2B&_targetURL=http%253A%252F%252Fwww.ncbi.nlm.nih.gov%252Fentrez%252Fquery.fcgi%253Fcmd%253Dsearch%2526db%253Dnucleotide%2526doptcmdl%253Dgenbank%2526term%253DNP_001007605%5Baccn%5D) | Swiss-Prot:[P05318](http://www.sciencedirect.com/science?_ob=RedirectURL&_method=externObjLink&_locator=genbank&_cdi=4941&_plusSign=%2B&_targetURL=http%253A%252F%252Fwww.ncbi.nlm.nih.gov%252Fentrez%252Fquery.fcgi%253Fcmd%253Dsearch%2526db%253Dnucleotide%2526doptcmdl%253Dgenbank%2526term%253DP05318%5Baccn%5D) |
| L3 | GenBank:AAM94270 | RefSeq:XP_791350 | GenBank:AAX48833 | Swiss-Prot:[O16797](http://www.sciencedirect.com/science?_ob=RedirectURL&_method=externObjLink&_locator=genbank&_cdi=4941&_plusSign=%2B&_targetURL=http%253A%252F%252Fwww.ncbi.nlm.nih.gov%252Fentrez%252Fquery.fcgi%253Fcmd%253Dsearch%2526db%253Dnucleotide%2526doptcmdl%253Dgenbank%2526term%253DO16797%5Baccn%5D) | RefSeq:[NP_942048](http://www.sciencedirect.com/science?_ob=RedirectURL&_method=externObjLink&_locator=genbank&_cdi=4941&_plusSign=%2B&_targetURL=http%253A%252F%252Fwww.ncbi.nlm.nih.gov%252Fentrez%252Fquery.fcgi%253Fcmd%253Dsearch%2526db%253Dnucleotide%2526doptcmdl%253Dgenbank%2526term%253DNP_942048%5Baccn%5D) | GenBank:[AAA88732](http://www.sciencedirect.com/science?_ob=RedirectURL&_method=externObjLink&_locator=genbank&_cdi=4941&_plusSign=%2B&_targetURL=http%253A%252F%252Fwww.ncbi.nlm.nih.gov%252Fentrez%252Fquery.fcgi%253Fcmd%253Dsearch%2526db%253Dnucleotide%2526doptcmdl%253Dgenbank%2526term%253DAAA88732%5Baccn%5D) |
| L7 | GenBank:AAN05591 | RefSeq:XP_792413 | GenBank:AAX48837 | Swiss-Prot:[P32100](http://www.sciencedirect.com/science?_ob=RedirectURL&_method=externObjLink&_locator=genbank&_cdi=4941&_plusSign=%2B&_targetURL=http%253A%252F%252Fwww.ncbi.nlm.nih.gov%252Fentrez%252Fquery.fcgi%253Fcmd%253Dsearch%2526db%253Dnucleotide%2526doptcmdl%253Dgenbank%2526term%253DP32100%5Baccn%5D) | Swiss-Prot:[P05426](http://www.sciencedirect.com/science?_ob=RedirectURL&_method=externObjLink&_locator=genbank&_cdi=4941&_plusSign=%2B&_targetURL=http%253A%252F%252Fwww.ncbi.nlm.nih.gov%252Fentrez%252Fquery.fcgi%253Fcmd%253Dsearch%2526db%253Dnucleotide%2526doptcmdl%253Dgenbank%2526term%253DP05426%5Baccn%5D) | Swiss-Prot:[Q12213](http://www.sciencedirect.com/science?_ob=RedirectURL&_method=externObjLink&_locator=genbank&_cdi=4941&_plusSign=%2B&_targetURL=http%253A%252F%252Fwww.ncbi.nlm.nih.gov%252Fentrez%252Fquery.fcgi%253Fcmd%253Dsearch%2526db%253Dnucleotide%2526doptcmdl%253Dgenbank%2526term%253DQ12213%5Baccn%5D) |
| L9 | GenBank:AAN05606 | RefSeq:XP_796514 | GenBank:AAX48839 | Swiss-Prot:[P50882](http://www.sciencedirect.com/science?_ob=RedirectURL&_method=externObjLink&_locator=genbank&_cdi=4941&_plusSign=%2B&_targetURL=http%253A%252F%252Fwww.ncbi.nlm.nih.gov%252Fentrez%252Fquery.fcgi%253Fcmd%253Dsearch%2526db%253Dnucleotide%2526doptcmdl%253Dgenbank%2526term%253DP50882%5Baccn%5D) | RefSeq:[NP_001007599](http://www.sciencedirect.com/science?_ob=RedirectURL&_method=externObjLink&_locator=genbank&_cdi=4941&_plusSign=%2B&_targetURL=http%253A%252F%252Fwww.ncbi.nlm.nih.gov%252Fentrez%252Fquery.fcgi%253Fcmd%253Dsearch%2526db%253Dnucleotide%2526doptcmdl%253Dgenbank%2526term%253DNP_001007599%5Baccn%5D) | PIR:[S53915](http://www.sciencedirect.com/science?_ob=RedirectURL&_method=externObjLink&_locator=genbank&_cdi=4941&_plusSign=%2B&_targetURL=http%253A%252F%252Fwww.ncbi.nlm.nih.gov%252Fentrez%252Fquery.fcgi%253Fcmd%253Dsearch%2526db%253Dnucleotide%2526doptcmdl%253Dgenbank%2526term%253DS53915%5Baccn%5D) |
| L11 | GenBank:AAN05587 | RefSeq:XP_786879 | GenBank:AAX48843 | Swiss-Prot:[P46222](http://www.sciencedirect.com/science?_ob=RedirectURL&_method=externObjLink&_locator=genbank&_cdi=4941&_plusSign=%2B&_targetURL=http%253A%252F%252Fwww.ncbi.nlm.nih.gov%252Fentrez%252Fquery.fcgi%253Fcmd%253Dsearch%2526db%253Dnucleotide%2526doptcmdl%253Dgenbank%2526term%253DP46222%5Baccn%5D) | Swiss-Prot:[P62914](http://www.sciencedirect.com/science?_ob=RedirectURL&_method=externObjLink&_locator=genbank&_cdi=4941&_plusSign=%2B&_targetURL=http%253A%252F%252Fwww.ncbi.nlm.nih.gov%252Fentrez%252Fquery.fcgi%253Fcmd%253Dsearch%2526db%253Dnucleotide%2526doptcmdl%253Dgenbank%2526term%253DP62914%5Baccn%5D) | Swiss-Prot:[P06380](http://www.sciencedirect.com/science?_ob=RedirectURL&_method=externObjLink&_locator=genbank&_cdi=4941&_plusSign=%2B&_targetURL=http%253A%252F%252Fwww.ncbi.nlm.nih.gov%252Fentrez%252Fquery.fcgi%253Fcmd%253Dsearch%2526db%253Dnucleotide%2526doptcmdl%253Dgenbank%2526term%253DP06380%5Baccn%5D) |
| L13 | GenBank:AAQ57120 | RefSeq:XP_791871 | GenBank:AAX48845 | PIR:[S42877](http://www.sciencedirect.com/science?_ob=RedirectURL&_method=externObjLink&_locator=genbank&_cdi=4941&_plusSign=%2B&_targetURL=http%253A%252F%252Fwww.ncbi.nlm.nih.gov%252Fentrez%252Fquery.fcgi%253Fcmd%253Dsearch%2526db%253Dnucleotide%2526doptcmdl%253Dgenbank%2526term%253DS42877%5Baccn%5D) | RefSeq:[NP_112363](http://www.sciencedirect.com/science?_ob=RedirectURL&_method=externObjLink&_locator=genbank&_cdi=4941&_plusSign=%2B&_targetURL=http%253A%252F%252Fwww.ncbi.nlm.nih.gov%252Fentrez%252Fquery.fcgi%253Fcmd%253Dsearch%2526db%253Dnucleotide%2526doptcmdl%253Dgenbank%2526term%253DNP_112363%5Baccn%5D) | Swiss-Prot:[Q12690](http://www.sciencedirect.com/science?_ob=RedirectURL&_method=externObjLink&_locator=genbank&_cdi=4941&_plusSign=%2B&_targetURL=http%253A%252F%252Fwww.ncbi.nlm.nih.gov%252Fentrez%252Fquery.fcgi%253Fcmd%253Dsearch%2526db%253Dnucleotide%2526doptcmdl%253Dgenbank%2526term%253DQ12690%5Baccn%5D) |
| L14 | GenBank:AAN05609 | RefSeq:XP_788935 | GenBank:AAX48847 | Swiss-Prot:[P55841](http://www.sciencedirect.com/science?_ob=RedirectURL&_method=externObjLink&_locator=genbank&_cdi=4941&_plusSign=%2B&_targetURL=http%253A%252F%252Fwww.ncbi.nlm.nih.gov%252Fentrez%252Fquery.fcgi%253Fcmd%253Dsearch%2526db%253Dnucleotide%2526doptcmdl%253Dgenbank%2526term%253DP55841%5Baccn%5D) | RefSeq:[NP_075238](http://www.sciencedirect.com/science?_ob=RedirectURL&_method=externObjLink&_locator=genbank&_cdi=4941&_plusSign=%2B&_targetURL=http%253A%252F%252Fwww.ncbi.nlm.nih.gov%252Fentrez%252Fquery.fcgi%253Fcmd%253Dsearch%2526db%253Dnucleotide%2526doptcmdl%253Dgenbank%2526term%253DNP_075238%5Baccn%5D) | Swiss-Prot:[P38754](http://www.sciencedirect.com/science?_ob=RedirectURL&_method=externObjLink&_locator=genbank&_cdi=4941&_plusSign=%2B&_targetURL=http%253A%252F%252Fwww.ncbi.nlm.nih.gov%252Fentrez%252Fquery.fcgi%253Fcmd%253Dsearch%2526db%253Dnucleotide%2526doptcmdl%253Dgenbank%2526term%253DP38754%5Baccn%5D) |
| L18 | EMBL:CAD91422 | RefSeq:XP_783919 | GenBank:AAX48850 | RefSeq:[NP_648091](http://www.sciencedirect.com/science?_ob=RedirectURL&_method=externObjLink&_locator=genbank&_cdi=4941&_plusSign=%2B&_targetURL=http%253A%252F%252Fwww.ncbi.nlm.nih.gov%252Fentrez%252Fquery.fcgi%253Fcmd%253Dsearch%2526db%253Dnucleotide%2526doptcmdl%253Dgenbank%2526term%253DNP_648091%5Baccn%5D) | RefSeq:[NP_112364](http://www.sciencedirect.com/science?_ob=RedirectURL&_method=externObjLink&_locator=genbank&_cdi=4941&_plusSign=%2B&_targetURL=http%253A%252F%252Fwww.ncbi.nlm.nih.gov%252Fentrez%252Fquery.fcgi%253Fcmd%253Dsearch%2526db%253Dnucleotide%2526doptcmdl%253Dgenbank%2526term%253DNP_112364%5Baccn%5D) | Swiss-Prot:[P07279](http://www.sciencedirect.com/science?_ob=RedirectURL&_method=externObjLink&_locator=genbank&_cdi=4941&_plusSign=%2B&_targetURL=http%253A%252F%252Fwww.ncbi.nlm.nih.gov%252Fentrez%252Fquery.fcgi%253Fcmd%253Dsearch%2526db%253Dnucleotide%2526doptcmdl%253Dgenbank%2526term%253DP07279%5Baccn%5D) |
| L21 | GenBank:AAN05604 | RefSeq:XP_783316 | GenBank:AAX48853 | RefSeq:[NP_610144](http://www.sciencedirect.com/science?_ob=RedirectURL&_method=externObjLink&_locator=genbank&_cdi=4941&_plusSign=%2B&_targetURL=http%253A%252F%252Fwww.ncbi.nlm.nih.gov%252Fentrez%252Fquery.fcgi%253Fcmd%253Dsearch%2526db%253Dnucleotide%2526doptcmdl%253Dgenbank%2526term%253DNP_610144%5Baccn%5D) | RefSeq:[NP_445782](http://www.sciencedirect.com/science?_ob=RedirectURL&_method=externObjLink&_locator=genbank&_cdi=4941&_plusSign=%2B&_targetURL=http%253A%252F%252Fwww.ncbi.nlm.nih.gov%252Fentrez%252Fquery.fcgi%253Fcmd%253Dsearch%2526db%253Dnucleotide%2526doptcmdl%253Dgenbank%2526term%253DNP_445782%5Baccn%5D) | Swiss-Prot:[Q02753](http://www.sciencedirect.com/science?_ob=RedirectURL&_method=externObjLink&_locator=genbank&_cdi=4941&_plusSign=%2B&_targetURL=http%253A%252F%252Fwww.ncbi.nlm.nih.gov%252Fentrez%252Fquery.fcgi%253Fcmd%253Dsearch%2526db%253Dnucleotide%2526doptcmdl%253Dgenbank%2526term%253DQ02753%5Baccn%5D) |
| L22 | GenBank:AAN05585 | RefSeq:XP_782165 | GenBank:AAX48854 | Swiss-Prot:[P50887](http://www.sciencedirect.com/science?_ob=RedirectURL&_method=externObjLink&_locator=genbank&_cdi=4941&_plusSign=%2B&_targetURL=http%253A%252F%252Fwww.ncbi.nlm.nih.gov%252Fentrez%252Fquery.fcgi%253Fcmd%253Dsearch%2526db%253Dnucleotide%2526doptcmdl%253Dgenbank%2526term%253DP50887%5Baccn%5D) | RefSeq:[NP_112366](http://www.sciencedirect.com/science?_ob=RedirectURL&_method=externObjLink&_locator=genbank&_cdi=4941&_plusSign=%2B&_targetURL=http%253A%252F%252Fwww.ncbi.nlm.nih.gov%252Fentrez%252Fquery.fcgi%253Fcmd%253Dsearch%2526db%253Dnucleotide%2526doptcmdl%253Dgenbank%2526term%253DNP_112366%5Baccn%5D) | Swiss-Prot:[P05749](http://www.sciencedirect.com/science?_ob=RedirectURL&_method=externObjLink&_locator=genbank&_cdi=4941&_plusSign=%2B&_targetURL=http%253A%252F%252Fwww.ncbi.nlm.nih.gov%252Fentrez%252Fquery.fcgi%253Fcmd%253Dsearch%2526db%253Dnucleotide%2526doptcmdl%253Dgenbank%2526term%253DP05749%5Baccn%5D) |
| L24 | EMBL:CAD91424 | RefSeq:XP_792367 | GenBank:AAX48857 | RefSeq:[NP_609649](http://www.sciencedirect.com/science?_ob=RedirectURL&_method=externObjLink&_locator=genbank&_cdi=4941&_plusSign=%2B&_targetURL=http%253A%252F%252Fwww.ncbi.nlm.nih.gov%252Fentrez%252Fquery.fcgi%253Fcmd%253Dsearch%2526db%253Dnucleotide%2526doptcmdl%253Dgenbank%2526term%253DNP_609649%5Baccn%5D) | RefSeq:[NP_071960](http://www.sciencedirect.com/science?_ob=RedirectURL&_method=externObjLink&_locator=genbank&_cdi=4941&_plusSign=%2B&_targetURL=http%253A%252F%252Fwww.ncbi.nlm.nih.gov%252Fentrez%252Fquery.fcgi%253Fcmd%253Dsearch%2526db%253Dnucleotide%2526doptcmdl%253Dgenbank%2526term%253DNP_071960%5Baccn%5D) | Swiss-Prot:[P04449](http://www.sciencedirect.com/science?_ob=RedirectURL&_method=externObjLink&_locator=genbank&_cdi=4941&_plusSign=%2B&_targetURL=http%253A%252F%252Fwww.ncbi.nlm.nih.gov%252Fentrez%252Fquery.fcgi%253Fcmd%253Dsearch%2526db%253Dnucleotide%2526doptcmdl%253Dgenbank%2526term%253DP04449%5Baccn%5D) |
| L28 | GenBank:AAX11340 | RefSeq:XP_789202 | GenBank:AAX48862 | Swiss-Prot:[Q9VZS5](http://www.sciencedirect.com/science?_ob=RedirectURL&_method=externObjLink&_locator=genbank&_cdi=4941&_plusSign=%2B&_targetURL=http%253A%252F%252Fwww.ncbi.nlm.nih.gov%252Fentrez%252Fquery.fcgi%253Fcmd%253Dsearch%2526db%253Dnucleotide%2526doptcmdl%253Dgenbank%2526term%253DQ9VZS5%5Baccn%5D) | RefSeq:[NP_073188](http://www.sciencedirect.com/science?_ob=RedirectURL&_method=externObjLink&_locator=genbank&_cdi=4941&_plusSign=%2B&_targetURL=http%253A%252F%252Fwww.ncbi.nlm.nih.gov%252Fentrez%252Fquery.fcgi%253Fcmd%253Dsearch%2526db%253Dnucleotide%2526doptcmdl%253Dgenbank%2526term%253DNP_073188%5Baccn%5D) | DDBJ:[BAA31554](http://www.sciencedirect.com/science?_ob=RedirectURL&_method=externObjLink&_locator=genbank&_cdi=4941&_plusSign=%2B&_targetURL=http%253A%252F%252Fwww.ncbi.nlm.nih.gov%252Fentrez%252Fquery.fcgi%253Fcmd%253Dsearch%2526db%253Dnucleotide%2526doptcmdl%253Dgenbank%2526term%253DBAA31554%5Baccn%5D) |
| L30 | GenBank:AAN05584 | RefSeq:XP_783150 | GenBank:AAX48864 | RefSeq:[NP_524687](http://www.sciencedirect.com/science?_ob=RedirectURL&_method=externObjLink&_locator=genbank&_cdi=4941&_plusSign=%2B&_targetURL=http%253A%252F%252Fwww.ncbi.nlm.nih.gov%252Fentrez%252Fquery.fcgi%253Fcmd%253Dsearch%2526db%253Dnucleotide%2526doptcmdl%253Dgenbank%2526term%253DNP_524687%5Baccn%5D) | RefSeq:[NP_073190](http://www.sciencedirect.com/science?_ob=RedirectURL&_method=externObjLink&_locator=genbank&_cdi=4941&_plusSign=%2B&_targetURL=http%253A%252F%252Fwww.ncbi.nlm.nih.gov%252Fentrez%252Fquery.fcgi%253Fcmd%253Dsearch%2526db%253Dnucleotide%2526doptcmdl%253Dgenbank%2526term%253DNP_073190%5Baccn%5D) | Swiss-Prot:[P14120](http://www.sciencedirect.com/science?_ob=RedirectURL&_method=externObjLink&_locator=genbank&_cdi=4941&_plusSign=%2B&_targetURL=http%253A%252F%252Fwww.ncbi.nlm.nih.gov%252Fentrez%252Fquery.fcgi%253Fcmd%253Dsearch%2526db%253Dnucleotide%2526doptcmdl%253Dgenbank%2526term%253DP14120%5Baccn%5D) |
| L31 | EMBL:CAD91431 | RefSeq:XP_793832 | GenBank:AAX48865 | Swiss-Prot:[Q9V597](http://www.sciencedirect.com/science?_ob=RedirectURL&_method=externObjLink&_locator=genbank&_cdi=4941&_plusSign=%2B&_targetURL=http%253A%252F%252Fwww.ncbi.nlm.nih.gov%252Fentrez%252Fquery.fcgi%253Fcmd%253Dsearch%2526db%253Dnucleotide%2526doptcmdl%253Dgenbank%2526term%253DQ9V597%5Baccn%5D) | RefSeq:[NP_071951](http://www.sciencedirect.com/science?_ob=RedirectURL&_method=externObjLink&_locator=genbank&_cdi=4941&_plusSign=%2B&_targetURL=http%253A%252F%252Fwww.ncbi.nlm.nih.gov%252Fentrez%252Fquery.fcgi%253Fcmd%253Dsearch%2526db%253Dnucleotide%2526doptcmdl%253Dgenbank%2526term%253DNP_071951%5Baccn%5D) | Swiss-Prot:[P04649](http://www.sciencedirect.com/science?_ob=RedirectURL&_method=externObjLink&_locator=genbank&_cdi=4941&_plusSign=%2B&_targetURL=http%253A%252F%252Fwww.ncbi.nlm.nih.gov%252Fentrez%252Fquery.fcgi%253Fcmd%253Dsearch%2526db%253Dnucleotide%2526doptcmdl%253Dgenbank%2526term%253DP04649%5Baccn%5D) |
| L32 | GenBank:AAN05611 | RefSeq:XP_782126 | GenBank:AAX48866 | RefSeq:[NP_733339](http://www.sciencedirect.com/science?_ob=RedirectURL&_method=externObjLink&_locator=genbank&_cdi=4941&_plusSign=%2B&_targetURL=http%253A%252F%252Fwww.ncbi.nlm.nih.gov%252Fentrez%252Fquery.fcgi%253Fcmd%253Dsearch%2526db%253Dnucleotide%2526doptcmdl%253Dgenbank%2526term%253DNP_733339%5Baccn%5D) | RefSeq:[NP_037358](http://www.sciencedirect.com/science?_ob=RedirectURL&_method=externObjLink&_locator=genbank&_cdi=4941&_plusSign=%2B&_targetURL=http%253A%252F%252Fwww.ncbi.nlm.nih.gov%252Fentrez%252Fquery.fcgi%253Fcmd%253Dsearch%2526db%253Dnucleotide%2526doptcmdl%253Dgenbank%2526term%253DNP_037358%5Baccn%5D) | Swiss-Prot:[P38061](http://www.sciencedirect.com/science?_ob=RedirectURL&_method=externObjLink&_locator=genbank&_cdi=4941&_plusSign=%2B&_targetURL=http%253A%252F%252Fwww.ncbi.nlm.nih.gov%252Fentrez%252Fquery.fcgi%253Fcmd%253Dsearch%2526db%253Dnucleotide%2526doptcmdl%253Dgenbank%2526term%253DP38061%5Baccn%5D) |
| L36 | GenBank:AAL99982 | RefSeq:XP_780348 | GenBank:AAX48870 | RefSeq:[NP_476629](http://www.sciencedirect.com/science?_ob=RedirectURL&_method=externObjLink&_locator=genbank&_cdi=4941&_plusSign=%2B&_targetURL=http%253A%252F%252Fwww.ncbi.nlm.nih.gov%252Fentrez%252Fquery.fcgi%253Fcmd%253Dsearch%2526db%253Dnucleotide%2526doptcmdl%253Dgenbank%2526term%253DNP_476629%5Baccn%5D) | RefSeq:[NP_071949](http://www.sciencedirect.com/science?_ob=RedirectURL&_method=externObjLink&_locator=genbank&_cdi=4941&_plusSign=%2B&_targetURL=http%253A%252F%252Fwww.ncbi.nlm.nih.gov%252Fentrez%252Fquery.fcgi%253Fcmd%253Dsearch%2526db%253Dnucleotide%2526doptcmdl%253Dgenbank%2526term%253DNP_071949%5Baccn%5D) | Swiss-Prot:[O14455](http://www.sciencedirect.com/science?_ob=RedirectURL&_method=externObjLink&_locator=genbank&_cdi=4941&_plusSign=%2B&_targetURL=http%253A%252F%252Fwww.ncbi.nlm.nih.gov%252Fentrez%252Fquery.fcgi%253Fcmd%253Dsearch%2526db%253Dnucleotide%2526doptcmdl%253Dgenbank%2526term%253DO14455%5Baccn%5D) |
| L37 | GenBank:AAL99981 | RefSeq:XP_796231 | GenBank:AAX48872 | RefSeq:[NP_573005](http://www.sciencedirect.com/science?_ob=RedirectURL&_method=externObjLink&_locator=genbank&_cdi=4941&_plusSign=%2B&_targetURL=http%253A%252F%252Fwww.ncbi.nlm.nih.gov%252Fentrez%252Fquery.fcgi%253Fcmd%253Dsearch%2526db%253Dnucleotide%2526doptcmdl%253Dgenbank%2526term%253DNP_573005%5Baccn%5D) | RefSeq:[NP_112368](http://www.sciencedirect.com/science?_ob=RedirectURL&_method=externObjLink&_locator=genbank&_cdi=4941&_plusSign=%2B&_targetURL=http%253A%252F%252Fwww.ncbi.nlm.nih.gov%252Fentrez%252Fquery.fcgi%253Fcmd%253Dsearch%2526db%253Dnucleotide%2526doptcmdl%253Dgenbank%2526term%253DNP_112368%5Baccn%5D) | Swiss-Prot:[P49166](http://www.sciencedirect.com/science?_ob=RedirectURL&_method=externObjLink&_locator=genbank&_cdi=4941&_plusSign=%2B&_targetURL=http%253A%252F%252Fwww.ncbi.nlm.nih.gov%252Fentrez%252Fquery.fcgi%253Fcmd%253Dsearch%2526db%253Dnucleotide%2526doptcmdl%253Dgenbank%2526term%253DP49166%5Baccn%5D) |
